# Gene Transcription and Splicing of T-Type Channels Are Evolutionarily-Conserved Strategies for Regulating Channel Expression and Gating

**DOI:** 10.1371/journal.pone.0037409

**Published:** 2012-06-15

**Authors:** Adriano Senatore, J. David Spafford

**Affiliations:** Department of Biology, University of Waterloo, Waterloo, Canada; Virginia Commonwealth University, United States of America

## Abstract

T-type calcium channels operate within tightly regulated biophysical constraints for supporting rhythmic firing in the brain, heart and secretory organs of invertebrates and vertebrates. The snail T-type gene, *LCa_v_3* from *Lymnaea stagnalis*, possesses alternative, tandem donor splice sites enabling a choice of a large exon 8b (201 aa) or a short exon 25c (9 aa) in cytoplasmic linkers, similar to mammalian homologs. Inclusion of optional 25c exons in the III–IV linker of T-type channels speeds up kinetics and causes hyperpolarizing shifts in both activation and steady-state inactivation of macroscopic currents. The abundant variant lacking exon 25c is the workhorse of embryonic Ca_v_3 channels, whose high density and right-shifted activation and availability curves are expected to increase pace-making and allow the channels to contribute more significantly to cellular excitation in prenatal tissue. Presence of brain-enriched, optional exon 8b conserved with mammalian Ca_v_3.1 and encompassing the proximal half of the I–II linker, imparts a ∼50% reduction in total and surface-expressed LCa_v_3 channel protein, which accounts for reduced whole-cell calcium currents of +8b variants in HEK cells. Evolutionarily conserved optional exons in cytoplasmic linkers of Ca_v_3 channels regulate expression (exon 8b) and a battery of biophysical properties (exon 25c) for tuning specialized firing patterns in different tissues and throughout development.

## Introduction

**Figure 1:**
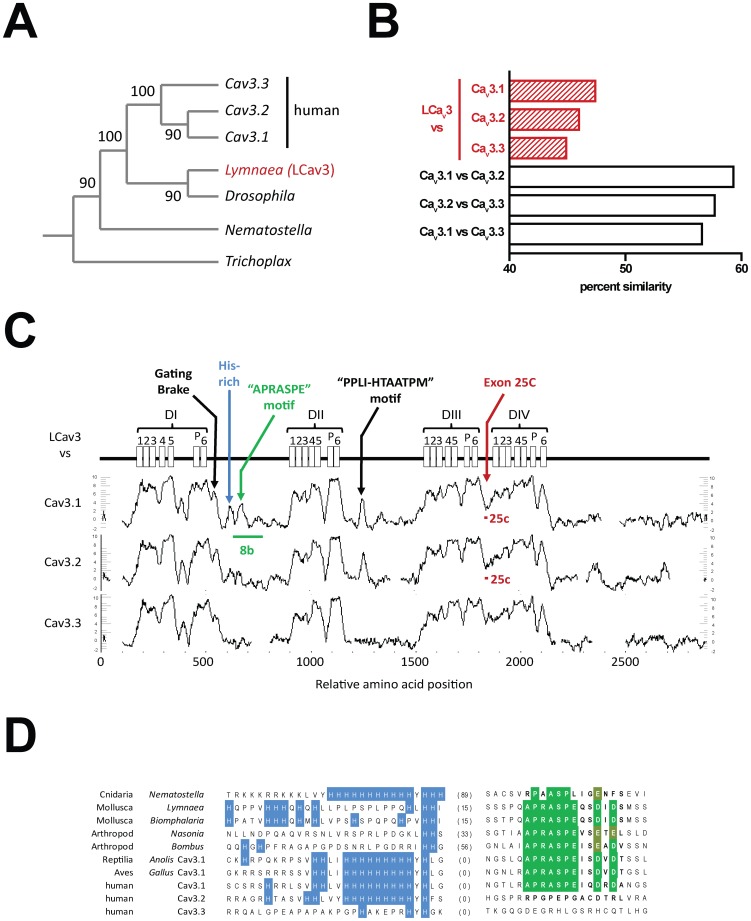
LCa_v_3 channel from snail is a distant homolog to mammalian Ca_v_3.1, Ca_v_3.2 and Ca_v_3.3, with conserved structural features in the cytoplasmic loops. (A) Phylogeny of T-type calcium channels using maximum parsimony with bootstrap scores indicated on branches. (B) Percent similarity of amino acid sequences of T-type channels (snail vs. human) using the Needleman-Wunsch global alignment algorithm. (C) Running average of similarity of snail versus human T-type channel amino acid sequences using a window of 25 aa (plotcon, EMBOSS http://emboss.open-bio.org/). (D) Alignment of T-type channel sequences in the I–II linker. Amino acids between the Histidine rich region and the APRASPE motif vary from 0 to 89 aa. Accession/IDs: (*Trichoplax* JGI:21513; *Nematostella* JGI:170705; *Drosophila* NM_001103419; *Lymnaea* AAO83843; *Biomphalaria* WUGI: Contig251; *Nasonia* XM_001604352; *Bombus* XM_003398839; *Anolis* XM_003217306; *Gallus* XM_001232653; human Ca_v_3.1 NP_061496.2; human Ca_v_3.2 NP_066921.2; human Ca_v_3.3 NP_066919.2).

Ca_v_3 channels are known for gating “transient” currents at low voltages near the resting membrane potential, and often depolarize cells to threshold in a cyclical manner to promote rhythmic firing (for reviews see Senatore *et al*., 2012 [1] and [2]). Animals with Ca_v_3 channels appear in relatives of extant multicellular organisms without tissues or organs (e.g. *Trichoplax*), and within Cnidarians, the simplest phlyum to harbor a nervous system (e.g. sea anemone *Nematostella*; **Figure 1** ***A***). Soft-bodied invertebrates, precursors to various animal phyla including molluscs, likely first appeared in the Late Vendian Period (650 to 543 mya), prior to the divergence of the single ancestral Ca_v_3 channel gene into three mammalian genes *CACNA1G* (Ca_v_3.1 or α_1_G), *CACNA1H* (Ca_v_3.2 or α_1_H) and *CACNA1I* (Ca_v_3.3 or α_1_I) [1]. Recently, we have cloned and expressed the first non-vertebrate Ca_v_3 channel *in vitro*, LCa_v_3, from the pond snail *Lymnaea stagnalis* [3]. Here, we describe two optional exons in the I–II and III–IV cytoplasmic linkers of *LCa_v_3* that are evolutionarily conserved with vertebrate Ca_v_3 channels and likely play critical roles in regulating membrane expression and an array of biophysical properties during development. The evolutionarily distant LCa_v_3 channel highlights key and fundamental features for T-type channels, providing an important perspective for understanding Ca_v_3 channel regulation.

## Results

### Structural conservation in Ca_v_3 channels

Comparisons between Ca_v_3 channels reveal that mammalian genes cluster more closely in overall sequence similarity amongst themselves than to the more evolutionarily distant and solitary *Ca_v_3* gene in snails (**Figure 1** **** ***A*** **,** **Figure 1** ***B***). Much of the divergence from the snail sequence lies in the tethered, cytoplasmic loops between transmembrane domains, which also bear surprisingly conserved islands of conservation (**Figure 1** ***C***). A signature helix-loop-helix in the proximal I–II cytoplasmic linker forms a “gating brake” that is unique to all Ca_v_3 channels, which when deleted augments characteristic features by shifting low-voltages of activation to even more hyperpolarized potentials, and increases kinetics of channel opening and closure [4], [5]. Downstream of the gating brake in vertebrate Ca_v_3.1 and invertebrate Ca_v_3 channels is a region that contains a large cluster of histidine residues, followed by an isolated and conserved “APRASPExxD/E” motif that is surrounded by unconserved sequences (**Figure 1** ***D***).

### Conserved splicing of exons 8b and 25c

**Figure 2:**
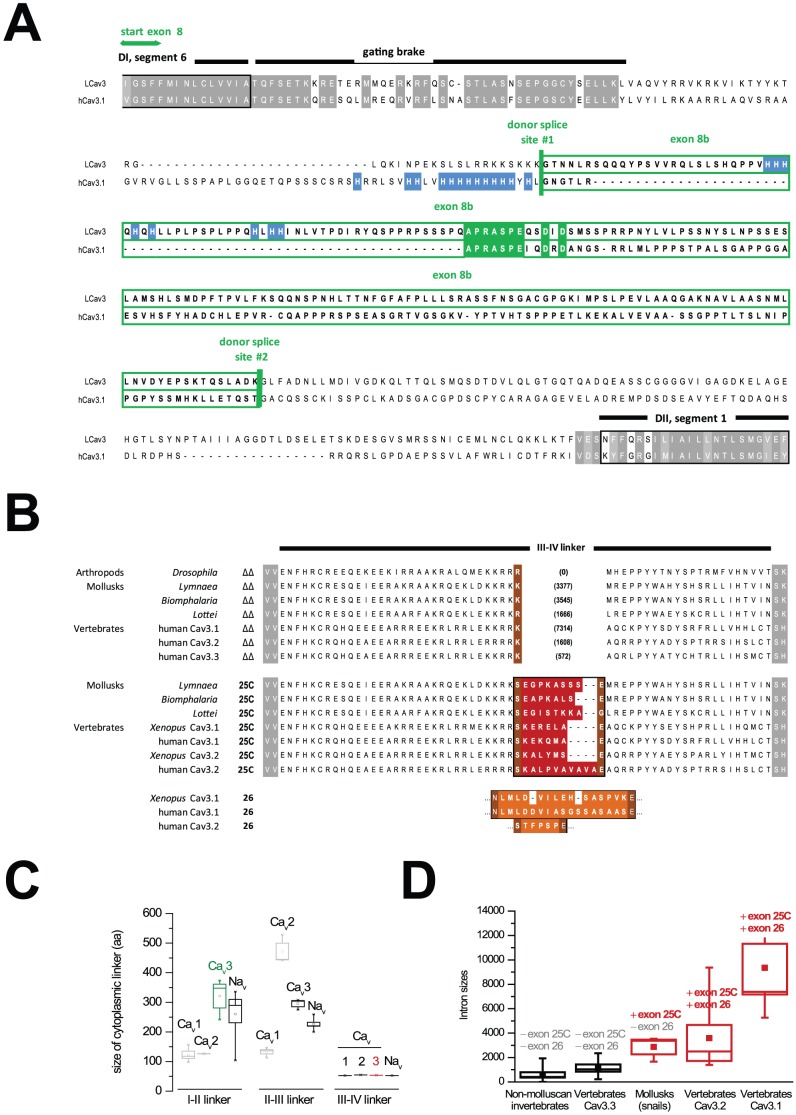
T-type calcium channels utilize alternative 5′ donor splice sites to generate optional exon 8b (I–II linker) and exon 25c (III–IV linker) isoforms. (A) Alignment of the snail LCa_v_3 and rat Ca_v_3.1 channel cytoplasmic I–II linkers, illustrating the conserved APRASPE motif in optional exon 8b. (B) Alignment of cytoplasmic III–IV linkers from invertebrates and vertebrates, illustrating the conservation of optional exon 25c and optional cassette exon 26. The numbers in brackets are the intron sizes at this position, ranging from 0 bp (*Drosophila*) to 7314 bp (human Ca_v_3.1). (C) Box plots showing size range distributions of cytoplasmic linkers from human Ca_v_1.1–1.4, Ca_v_2.1–2.3, Ca_v_3.1–3.3 and sodium channels Na_v_1.1–1.9 plus Na_v_X (mean +/− s.e.m.). (D) Box plot of intron sizes (mean +/− s.e.m.) for different T-type channels lacking or containing exon 25c and sometimes exon 26. Accession/IDs of genomic sequences: (*Drosophila* NM_001103419; *Biomphalaria* WUGI: Contig251; *Lottia* JGI: scaffold 59; *Xenopus* Ca_v_3.1 LOC100497283; *Xenopus* Ca_v_3.2 LOC100494894; human Ca_v_3.1 Gene ID: 8913; human Ca_v_3.1 Δ8b EF116283; human Ca_v_3.2 Gene ID: 8912; human Ca_v_3.3 Gene ID: 8911).

Interestingly, the “APRASPE” motif is contained within an optional portion of exon 8 that normally spans from Domain I segment 6 across ∼70% of the cytoplasmic I–II linker. The optional portion of exon 8 (termed exon 8b), is similarly spliced out in snail and mammalian [6] Ca_v_3 channel genes by use of alternative, upstream, intron donor splice sites within exon 8 that truncate the I–II linker coding sequences of *LCa_v_3* and *Ca_v_3.1* by 603 and 402 bp, respectively (**Figure S1)**. Exon 8b occurs downstream of the gating brake, and its omission shortens the I–II linker of LCa_v_3 channels by 201 aa (∼50%) and Ca_v_3.1 by 134 aa (∼39%; **Figure 2** ***A***). A second conserved region of alternative splicing corresponds precisely with the middle of the III–IV cytoplasmic linker (**Figure 2** ***B***), which is similarly short in closely related Na_v_ and Ca_v_ channels (54+/−1 aa; **Figure 2** ***C***). Splicing at alternative and more upstream, phase 1 intron donor splice sites shortens the III–IV linker by between 7 and 11 aa in different snail Ca_v_3 and mammalian Ca_v_3.1 and Ca_v_3.2 channels (**Figure 2** ***B*** **, Figure S2Figure S3**). *Ca_v_3.1* and *Ca_v_3.2* genes also possess downstream, similarly short optional cassette exons, termed exon 26, that code for between 6 and 19 aa, and that may be included in lieu of exon 25c or appear in tandem with it (**Figures 2** ***B*** **and Figure S4**). A consensus amino sequence or a number of positively or negatively charged residues is not a consistent feature of 25c exons. The only consistent feature is the first residue coding serine (coded by AGT) that completes the consensus kinase phosphorylation site (KKRKS for LCa_v_3), which also contributes to the consensus upstream 5′ donor splice site (GTRAGT; **Figures 2** ***B*** **and Figure S4**).

A simple evolutionary pattern has many invertebrate and mammalian *Ca_v_3.3* channel genes having “ΔΔ” isoforms lacking exons 25c or 26. Snail *LCa_v_3* possesses exon 25c besides ΔΔ, but analyses of over 48 independent RT-PCR products from snail embryonic and adult RNA did not uncover an optional cassette exon 26 for *LCa_v_3* (**Figure S5**). Vertebrate *Ca_v_3.1* and *Ca_v_3.2* possess both exons 25c and 26, and have correspondingly larger intron sizes spanning these regions than genes lacking either exon 26 (e.g. snail *Ca_v_3*), or both exons 25 and exons 26 (e.g. *Ca_v_3.3*; **Figure 2** ***D***). Larger intron sizes suggest more extensive regulation of alternative splicing for these short exons.

### LCa_v_3 channel expression patterns are consistent with those in mammals

**Figure 3:**
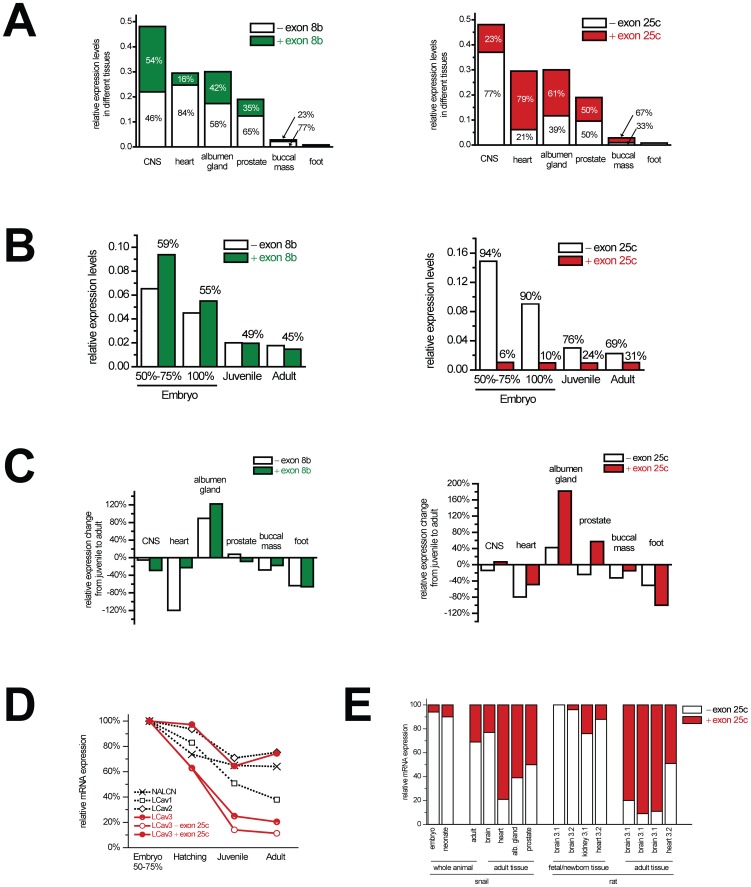
mRNA expression levels of LCa_v_3 and exon 8b and exon 25c splice variants, measured using quantitative RT-PCR. All expression values for (A) to (D) are standardized to *Lymnaea* HPRT1 control gene using the ΔΔCT method. (A) mRNA expression levels of LCa_v_3 (full bar values) and +/− exon 8b variants (left panel, green and white respectively) and +/− exon 25c variants (right panel, red and white respectively) in different adult snail organs. (B) mRNA expression levels of 8b (left panel) and 25c (right panel) LCa_v_3 variants in whole animals during different developmental stages (early embryo: 50–75%, near hatching embryo: 100%, juvenile and adult snails). (C) Change in mRNA levels of 8b and 25c variants in different snail organs from juvenile to adult. (D) Percent change in mRNA expression of snail cation channel genes. GenBank accession numbers: NALCN (AAO85435, AAO85436), LCa_v_1 (AF484079), LCa_v_2 (AF484082), LCa_v_3 (AF484084). (E) Percent of library clones or mRNA expression levels of snail and rat T-type channels with and without exon 25c. Besides snail, the other bar graphs include data gathered from other manuscripts, Fetal brain Ca_v_3.1 (n = 10, Latour *et al*., 2004 [9]), fetal brain Ca_v_3.2 (n = 53, Zhong *et al*., 2006 [12]), fetal kidney Ca_v_3.1 (n = 17, Monteil *et al*., 2000 [11]), neonatal heart Ca_v_3.2 (qPCR of 2 ug total RNA, David *et al*., 2010 [13]), adult brain Ca_v_3.1 (n = 10, Latour *et al*., 2004 [9]; n = 15, Monteil *et al*., 2000 [11]; n = 758, Emerick *et al*., 2006 [10]), heart Ca_v_3.2 (qPCR of 2 ug total RNA, David *et al*., 2010 [13]).

mRNA transcript levels measured by quantitative RT-PCR suggest that LCa_v_3 channels are most abundant in the brain, with intermediate expression in the heart and secretory glands (albumen and prostate), and almost non-detectable levels in skeletal-type muscle (buccal and foot musculature) of adult snails (**Figure 3** ***A***). The overall profile of expression closely matches that of the *Ca_v_3.1*, which is more abundant in the CNS, but not exclusively expressed in the adult brain (i.e. *Ca_v_3.3*), nor is it expressed more widely outside the brain (i.e. *Ca_v_3.2*) [7].

A general precipitous decline in mRNA transcript levels of *LCa_v_3* occurs from mid-embryo stage (50–75% embryonic development) to near hatching (100% embryonic development) to juvenile snails (**Figure 3** ***B*** **and** **Figure 3** ***D***), corresponding to a similar decline of mammalian *Ca_v_3* calcium channel gene expression during development (reviewed in Senatore *et al*., 2012 [1]). There is a continued, slight decline in Ca_v_3 channel expression from juvenile to adult animals except for a spike in expression in the albumen gland, likely associated with sexual maturation, and a dramatic decline in the heart, that also parallels the precipitous fall in *LCa_v_3* expression during mammalian heart development (**Figure 3** ***C***) [8].

### Conserved regulation of exon 8b and 25c splicing

*LCa_v_3* transcripts with and without 8b exons are of approximately equal abundance in the central nervous system and secretory glands (such as albumen gland; **Figure 3** ***A***), which approximates the findings in rat brain where there is significant mRNA expression of *Ca_v_3.1* with and without 8b exons [6]. We observe that the +8b isoform is less associated with the snail heart (16%) and buccal/foot musculature, compared to the higher levels in the brain and secretory glands (**Figure 3** ***A***). Exon 25c has a more striking developmentally-regulated pattern, with a precipitous decline in *LCa_v_3* transcripts lacking exon 25c from embryo to adults (**Figure 3** ***B*** **and** **Figure 3** ***D***), especially in the heart (**Figure 3** ***C***). The continued down-regulation of Ca_v_3 channels lacking exon 25c from juveniles to adults gives the appearance of a switch with an increasing relative expression of the plus exon 25c isoform in the adult brain and secretory albumen and prostate glands (**Figure 3** ***C***). Exon 25c-containing isoforms of *LCa_v_3* predominate in the adult heart (79%; **Figure 3** ***A***) and enhance in expression in most adult snail tissues (**Figure 3** ***C***). The relative absence of exon 25c before and at birth and its predominance in adults is consistent between snail *LCa_v_3* and mammalian *Ca_v_3.1* [9]–[11] and *Ca_v_3.2* [12], [13] (**Figure 3** ***E***). Correlative analysis of normalized qPCR data for plus and minus 8b and 25c variants, across all juvenile and adult tissues tested, reveals that +8b and −25c variants share similar variability in expression between the different tissues (correlation coefficient R^2^ = 0.885), and that −8b and +25c also share similar expression patterns (R^2^ = 0.898), while +8b/+25c and −8b/−25c have much lower correlation coefficients (**Figure S6**).

### Exon 25c selectively alters biophysical properties of LCa_v_3

**Figure 4:**
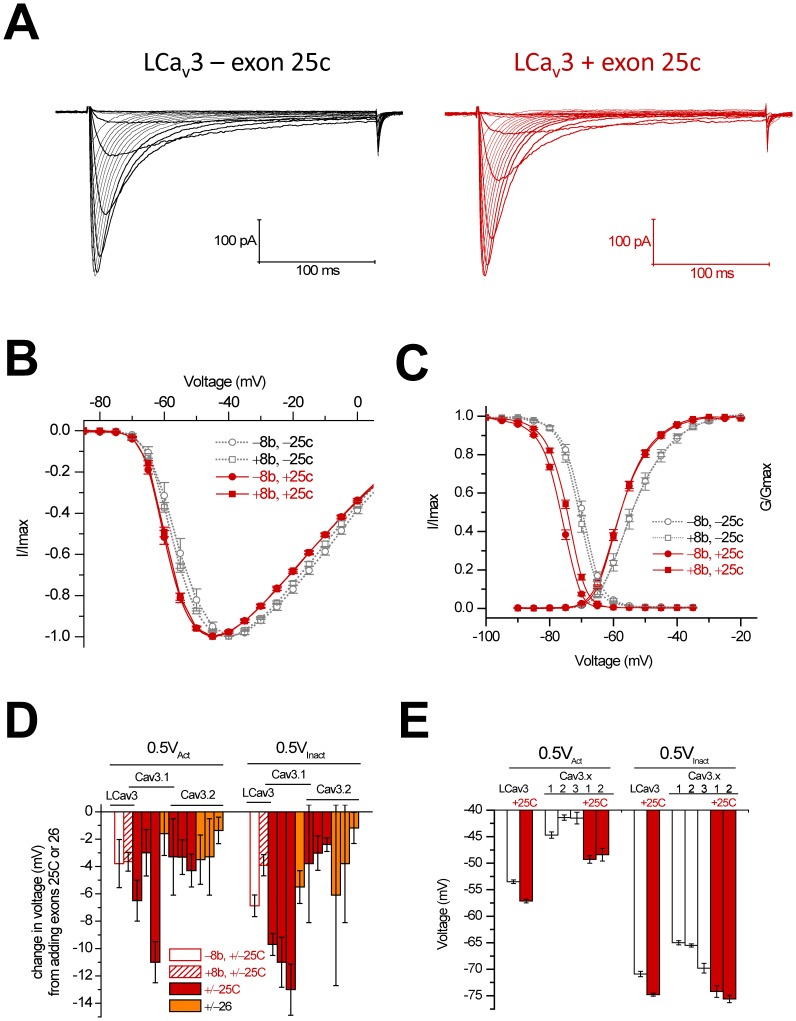
Changes in voltage-sensitivities of LCa_v_3 in response to the presence or absence of optional exons 8b and 25c. (A) Ensemble LCa_v_3 variant currents generated from a holding potential of −110 mV in 5 mV voltage steps from −90 mV to +10 mV. (B) Current-voltage curves for the four possible 8b and 25c variants (i.e. +8b −25c, −8b −25c, +8b +25c, and −8b +25c). (C) Combined activation and steady-state inactivation curves. (D) Changes in voltages of half-maximal activation (0.5V_Act_) and inactivation (0.5V_Inact_) associated with inclusion of exon 25c in LCa_v_3, compared to \ changes imposed by exons 25c and 26 in mammalian Ca_v_3.1 and Ca_v_3.2 reported in other manuscripts: Ca_v_3.1 exon 25: Chemin *et al*. 2001 [14]; Emerick *et al*. 2006 clone #25/clone #89 (-exon 14) [10]; Emerick *et al*., 2006, clone #153/clone #217 clones (+exon 14) [10]); Cav3.1 exon 26 (Chemin et al. 2001 [14]), Cav3.2 exon 25 Zhong et al. clone #513/clone #577 (+exon 35A) [10]; Ohkubo et al. 2005 [15], David et al. 2010 [13]), Cav3.2 exon 26 (Zhong et al 2006, clone #512/clone #544 (-exon 35A) [12]; Zhong et al. 2006, clone # 513/clone #545 (exon +35A) [12]; Ohkubo et al. 2005 [15]). (E) Voltages of half-activation and inactivation of LCa_v_3 resulting from inclusion or exclusion of exon 25c, compared to published data from mammalian studies. Ca_v_3.1 without exon 25c: Chemin *et al*. 2001 [14]; Ca_v_3.2 without exon 25c: David *et al*. 2010 [13]: Ca_v_3.1 and Ca_v_3.2 with exon 25c and Ca_v_3.3: Chemin *et al*., 2002 [31].

**Figure 5:**
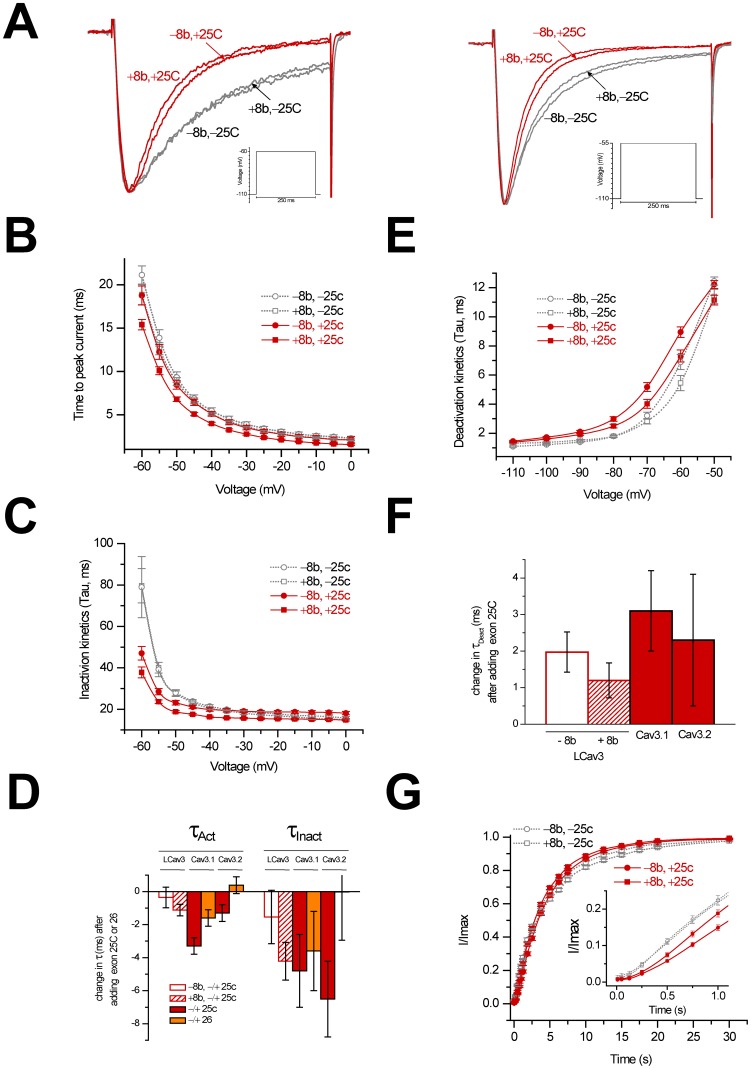
Changes in the kinetic properties of LCa_v_3 in response to the presence or absence of optional exons 8b and 25c. (A) Representative calcium current traces in response to voltage steps from −110 mV to −60 mV or −55 mV for the different cloned LCa_v_3 variants reveal stark changes in inactivation due to exon 25c. (B) Activation kinetics of LCa_v_3 variants measured by time to peak current reveal that exon 25c accelerates activation, which is most dramatic in the presence of exon 8b. (C) Tau curve fits of inactivation kinetics reveal a similar acceleration pattern attributable to exon 25c, especially near resting membrane potential, which is further enhanced by the inclusion of exon 8b. (D) Change in activation and inactivation kinetics of LCa_v_3 with and without exons 8b and 25c at steps to −40 mV (tau, ms), compared to changes documented for Ca_v_3.1 and Ca_v_3.2 associated with exons 25c and 26. Ca_v_3.1 exons 25c and 26: Chemin *et al*. 2001 [14]; Ca_v_3.2 exon 25 and 26, Ohkubo *et al*. 2005 [15]. (E) Deactivation kinetics (tau, ms) reveal that exon 25c slows down deactivation, especially at potentials more negative than rest (∼60 mV), while exon 8b accelerates deactivation between −70 and −60 mV. (F) Change in deactivation kinetics at steps to −70 mV (tau, ms) associated with 25c exons in LCa_v_3, Ca_v_3.1 (Chemin *et al*. 2001 [14]), Ca_v_3.2 (David *et al*. 2010 [13]). (G) Relatively slow recovery from inactivation for LCa_v_3 channel variants, from 2.5 ms to 6 s for full recovery (inset has 2.5 ms to 0.2 s timescale).

Using whole cell patch clamp technique we examined the biophysical consequences of the absence or presence of exons 8b and 25c in cloned LCa_v_3 variants heterologously expressed in HEK-293T cells, and performed one-way analysis of variance to asses statistical significance (**Table 1**). Surprisingly, the absence or presence of the large I–II linker 8b exon has comparatively little influence on the biophysical properties of LCa_v_3 (**Figures 4** **and** **Figure 5**) when compared to the small 25c insert. The peaks of the rapidly-activating and inactivating calcium currents were measured in response to 5 mV steps in the presence of 2 mM extracellular calcium (**Figure 4** ***A*** **and** **Figure 4** ***B*** **)**. Inclusion of exon 25c induces statistically significant −3.7 mV (+8b) and −3.8 mV (−8b) hyperpolarizing shifts in the half-maximal activation (V_0.5_) of LCa_v_3 calcium currents, extrapolated from the fitted Boltzmann of the plot of the fraction of maximal conductance at each voltage step (**Figure 4** ***C*** **and** **Table 1**). Exon 25c also causes parallel −3.9 and −6.9 mV hyperpolarizing shifts in half-maximal inactivation for +/− exon 8b LCa_v_3 variants, respectively (**Figure 4** ***C*** **and** **Table 1**). Boltzmann-fitted inactivation curves were generated by measuring residual peak currents at −35 mV following a series of inactivating, pre-pulse voltage steps (**Figure 4** ***C***). Similar hyperpolarizing shifts in the half maximal values for activation and inactivation curves are apparent for exon 25c inserts, regardless of the difference in their sequence or gene isoform type for snail LCa_v_3, or mammalian Ca_v_3.1 [10], [14] or Ca_v_3.2 channels [12], [13], [15] (**Figure 4** ***D*** **and** **Table 1**). The effect of exon 25c on the hyperpolarizing shift is greater than the differences in voltage-sensitivities between different T-type channels (**Figure 4** ***E***). Optional exon 26 also causes shifts in the voltage-sensitivities of activation and inactivation for Ca_v_3.1and Ca_v_3.2 (**Figure 4** ***D***), although generally the differences are much less dramatic than those imposed by exon 25c.

**Table 1:** Summary of biophysical parameters of LCav3 channel variants containing exons 8b and 25c expressed in HEK-293T cells, with one-way analysis of variance to assess statistical significance.

	LCa_v_3	LCa_v_3	LCa_v_3	LCa_v_3	Significance					
	+8b −25c	−8b −25c	+8b +25c	−8b +25c	A vs. B	A vs. C	A vs. D	B vs. C	B vs. D	C vs. D
	(A)	(B)	(C)	(D)						
**Activation**										
V_0.5_ (mV)	−53.48±0.34 (13)	−53.36±1.22 (6)	−57.13±0.35 (18)	−57.15±0.54 (14)	n.s	***	***	***	**	n.s.
Slope (mv)	5.46±0.14 (13)	5.45±0.20 (6)	4.34±0.09 (18)	4.60±0.14 (14)	n.s.	***	***	***	**	n.s.
**Inactivation**										
V_0.5_ (mV)	−70.89±0.49 (16)	−69.91±0.5 (14)	−74.80±0.28 (16)	−76.78±0.29 (15)	n.s.	***	***	***	***	***
Slope (mV)	2.93±0.08 (16)	3.11±0.10 (14)	3.12±0.05 (16)	3.14±0.04 (15)	n.s.	*	*	n.s.	n.s.	n.s.
**Activation kinetics**										
Rise −55 mV (ms)	12.62±0.70 (16)	13.87±0.96 (7)	10.09±0.44 (18)	12.25±0.86 (14)	n.s.	**	n.s.	***	n.s.	*
Rise −10 mV (ms)	2.35±0.12 (16)	2.58±0.23 (7)	1.75±0.09 (18)	2.32±0.26 (14)	n.s.	***	n.s.	***	n.s.	*
**Inactivation kinetics**										
τ −55 mV (ms)	40.07±2.56 (16)	39.20±3.46 (7)	23.64±0.87 (n18)	28.46±1.51 (14)	n.s.	***	***	***	**	**
τ −10 mV (ms)	15.67±0.56 (16)	16.81±0.74 (7)	15.10±0.43 (18)	18.51±0.79 (14)	n.s.	n.s.	**	*	n.s.	***
**Deactivation kinetics**										
τ −100 mV (ms)	1.37±0.05 (19)	1.22±0.04 (11)	1.60±0.05 (18)	1.72±0.06 (14)	*	***	***	***	***	n.s.
τ −60 mV (ms)	5.45±0.52 (19)	6.89±0.52 (11)	7.31±0.41 (18)	8.95±0.36 (14)	n.s.	**	***	n.s.	**	**
**Inactivation Recovery**										
% recovery at 50ms	4.98±0.46 (14)	4.62±0.50 (12)	2.82±0.19 (16)	2.26±0.13 (23)	n.s.	***	***	***	***	*
% recovery at 2000ms	81.82±0.93 (14)	84.69±1.33 (12)	88.67±0.63 (16)	86.87±0.61 (23)	n.s.	***	***	**	n.s.	*
T_0.5_ (ms)	908.04±27.05 (14)	822.04±32.92 (12)	817.90±27.03 (16)	949.48±34.77 (23)	*	*	n.s.	n.s.	*	*
**Channel Expression**										
Current density (pA/pF)	135.70±18.22 (13)	244.49±22.53 (13)	123.77±18.98 (13)	251.56±33.72 (13)	***	n.s.	**	***	n.s.	**

n.s. not significant;

* p<0.05;

** p<0.005;

*** p<0.001.

Exon 25c also promotes a significant speeding up of channel kinetics, most apparent in currents elicited by small voltage steps (−60 or −55 mV; **Figure 5** ***A*** **and** **Table 1**). Activation kinetics are significantly faster in the presence of exon 25c, especially when exon 8b is also present, as measured as the delay to time to peak current (**Figure 5** ***B***), as are inactivation kinetics, as measured by single exponential tau curve fits (**Figure 5** ***C***). A role of exon 25c in promoting faster channel activation and inactivation is common to both snail and mammalian Ca_v_3 channels (**Figure 5** ***D***). Deactivation kinetics are slowed by exon 25c, which corresponds to a slower rate of closure of Ca_v_3 channels from the open state, especially those currents elicited from voltage steps down to negative voltages such as resting membrane potential or more hyperpolarized than rest (−100 to −65 mV; **Figure 5** ***E***). A slowing of deactivation kinetics in the presence of exon 25c is also a shared feature of snail and mammalian [14], [15] Ca_v_3 channels (**Figure 5** ***F***). Exon 25c also promotes a slowing of the recovery rate from inactivation at the earliest time points of recovery (<0.2 seconds; **Figure 5** ***G*** **, inset and** **Table 1**), reminiscent to the slowing of inactivation promoted by exon 25c in mammalian Ca_v_3 channels [14]. It should be noted that LCa_v_3 is relatively slow to recover from inactivation compared to mammalian Ca_v_3 channels, even in the absence of exon 25c (**Figure 5** ***G***). Exon 8b can fine tune the biophysical changes imparted by exon 25c, such as influencing kinetics at depolarized potentials (i.e. above −45 mV; **Figure 5** ***B*** **,** **Figure 5** ***C*** **,** **Table 1**), or speeding up deactivation, more pronounced in the presence of exon 25c, near resting membrane potential (i.e. −60 to −70 mV; **Figure 5** ***E*** **and** **Table 1**). In summary, snails and mammals possess highly variable short 25c exons that exert near identical biophysical changes to Ca_v_3 channels during development.

### Exon 8b selectively alters LCa_v_3 expression

**Figure 6:**
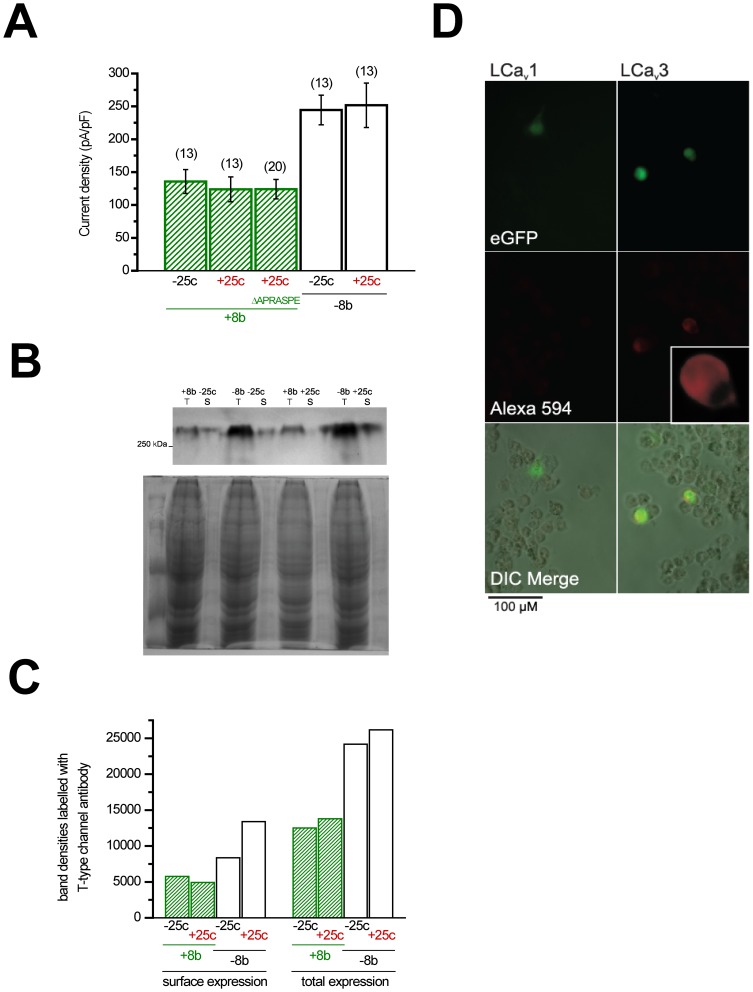
Changes in total and membrane expression of snail LCa_v_3 T-type channel in response to the presence or absence of optional exons 8b and 25c. (A) Current density (pA/pF) of peak calcium currents in the presence of optional exons (n = 13 for each variant), including a +8b −25c variant without the APRASPE_xx_D motif (n = 20). (B) Top: Western blot showing biotinylation-mediated fractioning of surface-expressed proteins, revealing that LCa_v_3 variants lacking exon 8b are more abundantly expressed in total protein extracts (T) of HEK-293T cells, and have corresponding increases in membrane expression (S). Bottom: Coomassie-stained replicate SDS-PAGE gel as the one used in the above blot, showing equal protein levels from the different HEK-derived total protein and surface protein extracts. (C) Densinometric quantification of bands visible on the Western blot indicates that −8b variants are ∼2-fold more abundant in both total protein and surface-expressed protein fractions. Notably, LCa_v_3 channel membrane expression is quite high in HEK cells (∼50% of total), regardless of splice variability in exons 8b and 25c. (D) Immunolabeling of HEK-293T cells transfected with pIRES2-EGFP expression constructs containing either LCa_v_3 (+8b −25c) or LCa_v_1 cDNA confirms specific membrane-delimited staining for LCa_v_3, with no labelling of LCa_v_1-transfected cells using α-LCa_v_3 polyclonal antibodies.

The large optional exon 8b has a major role likely associated with controlling the expression of Ca_v_3 channels, since transfection of equal molar quantities of cloned LCa_v_3 vectors into HEK-293T cells produces approximately 2-fold increases in current density recordings when variants lack 8b (**Figure 6** ***A*** **,** **Table 1**). In contrast, current density does not change in the absence or presence of exon 25c. Increased currents were also reported for mammalian Ca_v_3.1 lacking exon 8b [6], suggesting that analogous regulatory mechanisms might act on snail and mammalian 8b exons to control membrane expression. The only obvious similarity between exon 8b amino acid sequences of LCa_v_3 and Ca_v_3.1 is an APRASPE motif (**Figure 1** ***D***), which, when deleted, surprisingly has no effect on LCa_v_3 channel current density (**Figure 6** ***A***).

The doubling of current densities in the absence of exon 8b, attributable to an increase in the number of channels present at the membrane, could arise from increases in total protein expression or strictly increased trafficking to the membrane [6]. A possible change in membrane trafficking of LCa_v_3 associated with exon 8b was assessed by separating and quantifying biotinylated, membrane-delimited channel variants expressed in HEK-293T cells, relative to channels present in whole cell fractions on immunoblots labeled with polyclonal LCa_v_3 antibodies [16]. Antigen specificity of the polyclonal antibodies for these experiments was confirmed by immunolabeling of HEK cells transfected with either the T-type channel cDNA (+8b −25c), or that of snail LCa_v_1 calcium channel (**Figure 6** ***D***), and further tested on Western blots using expressed and purified LCa_v_3 I–II linker peptides (**Figure S7**). Biotinylation experiments revealed dramatic increases in both total and membrane-expressed fractions of transfected LCa_v_3 variants lacking exon 8b (**Figure 6** ***B*** **and** **Figure 6** ***C***), indicating that the doubling of current densities is likely due to an increase in protein expression, and not specifically increased membrane trafficking. We also inserted hemagglutinin (HA)-epitope tags in the extracellular Domain I s5–s6 loop of LCa_v_3 variants, to quantify the chemiluminescent signals of labeled, membrane-delimited epitope by luminometry [6]. Surprisingly, luminometry experiments were inconclusive, since there was a doubling of signal in permeabilized transfected cells compared to non-permeabilized conditions, regardless of the treatment, including cells transfected with untagged LCa_v_3 (i.e. +8b +25c) (**Figure S8**). In addition, all HA-tagged channels, regardless of their insert, produced <20-fold smaller recordable currents than their wild type channel counterparts (**Figure S9**).

## Discussion

### Evolution and development of Ca_v_3 channels

Invertebrates possess only a single Ca_v_3 channel gene which provides a reference point for evaluating fundamental features of T-type channels. We illustrate here two highly conserved and developmentally regulated optional exons in cytoplasmic linkers shared between invertebrates and mammals, that provide insights into the fundamental roles that alternative splicing has played in the early evolution of Ca_v_3 channels.

Ca_v_3 channels likely first appeared in early multicellular organisms, since single-celled animals, such as the coanoflagellates, have a single calcium channel homolog, an L-type (Ca_v_1), but no Ca_v_3 channel [1]. Likely, gene duplication of the L-type calcium channel gene generated a synaptic Ca_v_2, “N-type like” channel gene and a Ca_v_3 channel gene, in a close ancestor of primitive multi-cellular organisms (i.e. *Trichoplax*) or within an animal phylum with the most primitive nervous system, the Cnidarians, which both possess a full complement of single Ca_v_1, Ca_v_2 and Ca_v_3 channel genes (**Figure 1** ***A***; [17]). The snail homolog from *Lymnaea stagnalis* closely matches the mammalian Ca_v_3.1 and Ca_v_3.2 channels in the quintessential features of Ca_v_3 channels, which appear optimized for generating rhythmic firing patterns, with a low voltage range of gating and rapid kinetics to drive membrane depolarization from resting membrane potential to threshold quickly, and a property of slow deactivation kinetics, which keeps Ca_v_3 channels open to maximize their effectiveness, if they are not in a refractory, inactivated state [1]. Conserved features in invertebrates also extend to their developmental expression profile. Ca_v_3 channel mRNA levels fall precipitously (80%) from embryo to juvenile snails in comparison to a more gradual decline of related channels, such as Ca_v_2 and NALCN (∼20%) or L-type calcium channels (60%) (**Figure 3** ***D***). This decline in Ca_v_3 channel transcripts continues from juvenile to adults in most tissues and is most dramatic in the heart compared to the brain. The higher density of Ca_v_3 expression correlates well with the faster embryonic heart rate, and drops sharply with the slower heart rate after the rapid phase of growth (embryo/neonate to juvenile animals). Ca_v_3 channel expression is also highest in animals of smaller sizes [8], which have faster heart and metabolic rates associated with allometric scaling. A prominent Ca_v_3 current remains in adult snails [18], but this is diminished in the pace-making cells of increasingly large mammals, to a level that may be imperceptible in the adult human heart [8]. In the tissues we studied, only albumen and prostate glands, which grow dramatically in size from juvenile to adult snails, exhibit increases in Ca_v_3 channel expression, consistent with organ maturation and emergent properties for Ca_v_3 channels in secretory roles of sexually-mature animals.

A high embryonic level of Ca_v_3 channel expression is associated with expanded roles in the early proliferative states such as myoblast fusion [19], and recapitulated to high levels in disease states such as cancer [20] and ventricular hypertrophy [8]. Extracellular calcium contributes to contraction in immature muscle, which lacks the elaborate calcium delivery system in adult muscle involving transverse tubules signalling to intracellular calcium release units via coupling to membranal L-type calcium channels [21]. Adult invertebrate muscle is also primitive, lacking tetradic organization and striations [21], where Ca_v_3 channels can serve as the only calcium source for muscle contraction (*Polyorchis* jellyfish muscle) [22], are a major contributor to muscle action potentials (nematode) [23], provide an alternative to sodium spikes in giant motor neurons (*Aglantha* jellyfish) [24] or are a prominent source of calcium for the adult heart [18].

### Evolution of cytoplasmic linkers

The fall in mRNA expression from embryo to adult snails is almost exclusively with Ca_v_3 channels that lack exon 25c in the cytoplasmic III–IV linker (**Figure 3B**), leading to a change in mode of T-type channel activity in the transition from embryo to adult. Remarkably, III–IV linkers have been restricted to a discrete size of 54+/−1 aa in all human calcium and sodium channels, whereas the other cytoplasmic linkers substantially vary in size ranging from ∼100 to >500 aa (**Figure 2** ***C***). The shortness constrains the III–IV linker so that it is tightly coupled to the cytoplasmic end of the pore, preventing it from protruding too deeply into it. The III–IV linker is a primary agent for fast inactivation of Na_v_ channels, serving as a manhole cover with a hydrophobic latch (IFM) flanked by an alpha helix on either side that pivots on a flexible hinge to occlude the cytoplasmic pore [25]. The “inactivation particle” of the III–IV linker of Na_v_ channels is conserved down to single-celled coanoflagellates, is absent in all Ca_v_ channels [17], but a parallel role is likely played by the similar sized III–IV linker of Ca_v_3 channels. Evolution of an alternative 5′ donor splice site expands the middle of the III–IV linker to retain intron sequence as a 7 to 11 aa coded exon, extending exon 25 (dubbed exon 25c) in molluscs and vertebrate Ca_v_3.1 and Ca_v_3.2 channels beyond the tightly-regulated size of 54+/1 aa. Corresponding with III–IV linker exon variants, there is an added complexity of the regulation of alternative splicing, with a dramatic increase in intron size from non-molluscan invertebrates and vertebrate Ca_v_3.3 channel genes that lack optional exons in the III–IV linker, to the molluscan Ca_v_3 channel with an exon 25c, to vertebrate Ca_v_3.1 and Ca_v_3.2 channels which contain two differentially-regulated optional exons 25c and 26 (**Figure 2** ***D***). Splicing factors in spliceosomes have varying compositions in different developmental stages and cell types, to generate unique mRNAs from heteronuclear pre-mRNA with the guidance of specific nucleotide sequences within introns and adjacent exons [26]. Larger intron sizes suggest extensive regulation of alternative splicing for these short exons in different tissues [26]. Interestingly, +8b and −25c variants have a significantly correlated expression pattern amongst the juvenile and adult tissues tested by qPCR (correlation coefficient R^2^ = 0.885), as do −8b and +25c (R^2^ = 0.898), while +8b/+25c and −8b/−25c have lower R^2^ values (**Figure S6**). This suggests that there is a co-ordinated splicing of LCa_v_3 isoforms containing either +8b with −25c, or −8b with +25c in different tissues, and that these are the most physiologically relevant isoforms in juvenile and adult snails. However, alternative splicing can be somewhat stochastic [27], [28], and given the presence of all four splice variants together in many different tissues (**Figure 3**), it is probable that all four possible configurations are present in the animal, at least to some degree.

### Biophysical consequences of 25c and 26

Modeling studies suggest that there are general truisms associated with Ca_v_3 channel splicing, although Ca_v_3 channel behavior will vary considerably with background cellular context such as other ionic conductance and the resting membrane potential, as well as with subcellular localization [29]. Exon 25c imparts a hyperpolarizing shift in the activation and inactivation curves of Ca_v_3 channels (by a few to 10 mV), that restricts their activity because they are unavailable and inactivated at rest. If the membrane potential does not change significantly throughout development, enrichment of exon 25c in adults would serve to dampen the contribution of these channels to excitability. These variants would be more adept at driving post-inhibitory rebound excitation after strong hyperpolarizing input, which together with the faster activation and inactivation kinetics reported for plus exon 25c variants, would generate calcium spikes with a faster onset and faster attenuation after hypepolarization, reminiscent of low threshold spikes (LTS) in thalamocortical neurons [30]. Within action potential bursts that sometimes ride over LTS, the depolarizing contribution of Ca_v_3 calcium currents during action potential repolarization is maximized by the long delay of channel closure from the open state (slower deactivation kinetics) imparted by exon 25c [14], [31]. Exon 25c, the more prominent isoform in adults, promotes inactivation to limit excitability, and also slows the recovery from complete inactivation at the earliest time point of recovery. Given that Ca_v_3 channels play important roles in setting the rhythmicity of oscillatory firing, the above features suggest that the enrichment of exon 25c in adults, along with the developmental down-regulation of Ca_v_3 channel expression, serve to slow down oscillatory firing or diminish the contribution of Ca_v_3 channels to excitability in general.

Embryonic Ca_v_3 channels lacking exon 25c have properties more similar to Ca_v_3.3, with slower activation and inactivation kinetics, as well as right-shifted activation and steady-state inactivation curves [31]. ΔΔ variants conduct less calcium into the cell during a single burst, and are better suited for prolonged spiking at higher frequencies (e.g. burst firing in nRT neurons [26], [32]) due to a lower propensity for cumulative inactivation during high frequency firing. Rather than dampen, Ca_v_3.3 channel currents actually facilitate through the first few spikes in a train. Since ΔΔ variants are the predominant isoform in the embryo of both snails and mammals, the default state is likely one which favours a contribution of Ca_v_3 channels to more rapid firing patterns in embryonic cells relative to adult ones. In addition, Ca_v_3 channels without exon 25c are less inactivated at rest, with a more positive-shifted activation and inactivation curves, and are more readily available to open in response to depolarization, rather than relying on hyperpolarizing input. Ca_v_3.3 channels generally have a brain-specific, somato-dendritic localization where they extend further into the dendritic arbour than Ca_v_3.1 and Ca_v_3.2 [33]. Dendritic T-type channels are implicated in synaptic integration, where they serve to amplify post-synaptic inputs to the soma [34], and the slower activation and inactivation kinetics of Ca_v_3.3 might facilitate this role by allowing the channels to overcome the high input resistance of dendrites. Interestingly, modelling suggests that dendritic T-type channels are subject to a hyperpolarizing shift in their activation during somatic depolarization [29], which for Ca_v_3.3 channels would approximate their depolarized activation curves to match those of the more hyperpolarized and somatic Ca_v_3.1 and Ca_v_3.2.

Exon 25c sets the framework for the major biophysical differences between Ca_v_3 channels. Snail LCa_v_3 and mammalian Ca_v_3.1 and Ca_v_3.2 utilize exon 25c to differentiate themselves from Ca_v_3.3, with shifted activation and inactivation gating to significantly more hyperpolarized potentials, not achievable by merely switching to the expression of a different mammalian channel gene (i.e. Ca_v_3.1, Ca_v_3.2 or Ca_v_3.3; **Figure 4** ***E***). Indeed, systematic replacement of different trans-membrane and cytoplasmic regions of Ca_v_3.1 into Ca_v_3.3 creates chimeric channels that resemble Ca_v_3.1 only when an exon 25c-containing III–IV linker is inserted into Ca_v_3.3 [35]. These changes (measured in *Xenopus* oocytes) include dramatically shifted activation and inactivated curves in the hyperpolarizing direction (−9.5 mV and −8.6 mV, respectively), and increases in the kinetics of activation and inactivation [35].

### Structure of exon 25c and exon 26

Sequence comparisons do not elucidate a consistent picture for the structural requirements of exon 25c, since the number of charged residues can vary even amongst closely related species (e.g. freshwater and sea snail Ca_v_3 channels; *Lymnaea* SEGPKASSSE vs. *Lottia* SEGISTKKAG), size can vary considerably (e.g. between frog and human Ca_v_3.2 channels; SKALPVAVAVAE vs. SKALYMSE respectively) and a requirement for the first position being a serine residue to completing a consensus phosphorylation site is lacking when comparing human exon 25c and 26 inserts (SKEKQMA vs NLMLDDVIASGSSASAASE respectively; **Figure 2** ***B***). An effective, threshold size of 8 aa may be required for III–IV inserts, since the slightly shorter exon 26 insert of Ca_v_3.2 channels (STFPSPE), imparts only small biophysical changes such as a shift in the curve for steady-state inactivation (**Figure 4** ***D***), an no significant changes in kinetics of activation and inactivation (**Figure 5** ***D***). A role for residue charge would be consistent with Na_v_ channels, where clusters of charged residues in the III–IV linker differentially regulate the kinetics of fast inactivation [36].

### Role of exon 25c in gating

Dynamic clamp simulations suggest that minimal changes in the biophysical properties of the Ca_v_3 channel currents, especially in the voltage range corresponding to the base of the current-voltage curve, drastically alter the contribution of Ca_v_3 channels to calcium spikes [30], such as the low threshold calcium spikes in the thalamus characterized in states such as physiological sleep or pathological states such as epilepsy. LTS are often crowned with sodium channel-dependent action potential spike trains, whose frequency and longevity highly depends on the shape of the underlying calcium spike and thus Ca_v_3 channel activity [2]. While the suite of biophysical changes associated with inclusion of exon 25c provides Ca_v_3.1 and Ca_v_3.2 channels for fast post-inhibitory depolarizing responses with calcium spikes crowned by relatively fast but short lived spike trains (e.g. thalamocortical LTS), omission of exon 25c (and 26) creates variants better suited for LTS with a more delayed onset, less dependent on hyperpolarization, and crowned by longer lasting sodium channel spike trains (e.g. nRT LTS) [31]. Clearly even subtle differences may drastically influence the contribution played by Ca_v_3 channels under different conditions. These include differences in the channel genes (e.g. recovery from inactivation of Ca_v_3.1 vs Ca_v_3.2), other splice variants that modulate gating and trafficking (such as exons 8b and 38b for Ca_v_3.1, exon 35a for Ca_v_3.2, and exon 14 for Ca_v_3.1/Ca_v_3.2) [10], [12], [14], G protein modulation and phosphorylation (largely a capacity of Ca_v_3.2 channels [37]), potentiation by glutamate receptors (Ca_v_3.1 channels in cerebellar Purkinje fibers) [38], association with K^+^ channels [39], as well as being influenced by the shape of the trigger (e.g. synaptic input) [30].

### Role of exon 8b in expression

Exon 8b has only a minor influence on biophysical properties, while its omission dramatically increases the membrane expression of snail LCa_v_3 and mammalian Ca_v_3.1 (by ∼2-fold) in transfected human cells (**Figure 6** ***A***). A conserved APRASPE motif is an obvious, shared feature of exon 8b in invertebrate and Ca_v_3.1 channels, but its absence has little effect on the current densities of snail LCa_v_3 in mammalian cell lines. It may indicate that the APRASPE motif is not critical for protein and/or membrane expression, or that its function depends on tissue-specific factors not present in HEK-293T cells. Exon 8b is abundant in the snail brain and secretory organs and mostly lacking in the heart. It is contained in the largest cytoplasmic linker of Ca_v_3 and Na_v_ channels (**Figure 2** ***C***), which is also the primary domain for regulating expression of all Ca_v_ and Na_v_ channels. Expression of closely related high voltage-activated (HVA) Ca_v_1 and Ca_v_2 channels is promoted by assembly with an accessory β subunit binding to the proximate I–II linker (in the homologous position of the gating brake of Ca_v_3 channels) [40]. Increases in the expression of HVA channels have recently been shown to depend on the β subunit interaction that protects the channels from targeted degradation via the endoplasmic reticulum-associated protein degradation (ERAD) pathway [40]. It has been proposed that misfolding in the I–II linker of Ca_v_2.1 is responsible for pathological ER retention and ERAD of mutant channels associated with episodic ataxia [41].

Subunit assembly with accessory subunits is not a likely control mechanism for Ca_v_3 channel expression or protein stability, but the I–II linker of snail and mammalian Ca_v_3 channels is a key region for its regulation. Large deletions in the I–II linker (downstream of the gating brake) for snail LCa_v_3 and mammalian Ca_v_3.1, Ca_v_3.2 and Ca_v_3.3 moderately enhances, moderately enhances, dramatically enhances, and depresses membrane expression respectively [42]. Lower current densities observed in the presence of exon 8b could be attributed to regulation of channel trafficking to the cell membrane, or the depression of total protein expression of Ca_v_3 channels, or a combination of these factors. Our results indicate that inclusion of exon 8b, regardless of the presence or absence of exon 25c, causes dramatic decreases in both total protein *and* membrane expression of LCa_v_3 channels (**Figure 6** ***B*** **and** **Figure 6** ***C***).

### Conclusions from snail work

Work with snails provides a unique perspective for defining the fundamental features of Ca_v_3 channels. We show that the snail and mammalian channels operate within tightly regulated biophysical constraints for supporting rhythmic firing in the brain, heart and secretory organs, and that there are many remarkable parallels in expression patterns between respective Ca_v_3 channels and exon 8b and 25c splice isoforms. The presence of exon 25c in the III–IV linker, a region whose length is highly invariable in sodium and calcium channels of the 4-domain superfamily, suggests that this locus was exploited during T-type channel evolution to provide splice variants with markedly different biophysical properties. We suggest that the snail channel is more akin to Ca_v_3.1 because of the common regulation of membrane expression with exon 8b, which is enriched in the brain of both snails and mammals. If indeed Ca_v_3.1 is more reminiscent of the ancestral channel type, then Ca_v_3.2 deviated from Ca_v_3.1 less in biophysical terms, but rather in its greater capacity for modulation. Ca_v_3.3 became the most divergent of all T-type channels with a restricted tissue expression profile in the brain, and lacking an exon 25c. In the embryo, T-type channels are highly abundant and lack exon 25c, which supports accelerated rhythmic firing, and high channel density might also serve expanded roles such as the calcium delivery for contraction of immature muscle. In adults, there is an upregulation of T-type channels in secretory glands, coinciding with sexual maturation and active secretion of vesicular components into reproductive tracts. Further insights into T-type channels are facilitated in snails which have only a single Ca_v_3 channel gene, and a highly tractable and accessible preparation for studying its associated functions in brain, heart and secretory organs.

## Materials and Methods

Expanded materials and methods are provided as a supplement (**Methods S1**).

### Source of animals

Giant pond snails, *Lymnaea stagnalis* were raised in-house in a snail vivarium and breeding facility in B1-177, Department of Biology, University of Waterloo.

### Identification of splice variants

During the initial sequencing and cloning of LCa_v_3 [3], multiple variable sequences were identified, including conserved optional exons 8b in the I–II linker and 25c in the III–IV linker. PCR-screening attempts with *Lymnaea* cDNAs did not reveal exon 26, found in the III–IV linkers of mammalian Ca_v_3.1 and Ca_v_3.2 channel genes (**Figure S5**). Genomic sequence surrounding optional exons 8b and exon 25c were obtained by PCR and compared to other available snail and mammalian sequences (**Figures S1, Figure S2, Figure S3,** **Table 2**). PCR was also used to generate the APRASPEQSD sequence deletion, HA-tagged channels and −/+ exon 8b and −/+ exon 25c splice variants (**Table 2**).

**Table 2:** Primers used for sequencing and cloning.

Name of DNA primer	DNA primer sequence
III–IVL RT-PCR screen 5′1	CTTTCATCTCTCCGTCCTATTGGC
III–IVL RT-PCR screen 3′1	CTCTAATGTATCGGTAAAAGCCCAGTGC
III–IVL RT-PCR screen 5′2	CAAATCGGAGCCAATGTGAGGCAG
III–IVL RT-PCR screen 3′2	GCCAAATCAAAGTATTTGCTATTGATC
I–IIL intron 5′1	CTGTGCTCGCAGCATCTAATATGCTG
I–IIL intron 3′1	CTGAGTCTGGCCTGTGCCAAGCTGTA
I–IIL intron 5′2	CTCAATGTGGATTATGAGCCTAGCAAAACTCA
I–IIL intron 5′2	CATCTGTGTCACTCTGCATGGACAACTGTG
III–IVL intron 5′1	CGGGAGTCTCAAGAAATTGAGGAG
III–IVL intron 3′1	GCCAAATCAAAGTATTTGCTATTGATC
III–IVL intron 5′2	GAGGAGCGGGCCAAGCGTGCTGCT
III–IVL intron 3′2	TGTGTGAATGAGCAGACGGCTGTG
pGEM Adaptor 5′	CATG GCTCGAGATAGATCTATATGTCGACATCAAAA
pGEM Adaptor 3′	CGCG TTTTGATGTCGACATATAGATCTATCTCGAGC
LCav3 −8b +5P 5′	P-GCTTTTTGCTGACAATCTTCTTATGGAT
LCav3 −8b +5P 3′	P-CCTTTCTTTTTAGACTTTTTCCTGCGC
LCav3 +25c 5′	AATTGGGCCCGACGTCGCATG
LCav3 +25c 3′1	CGGACGACGATGCTTTAGGTCCCTCACTCTTTCTTTTTTTATCCAGTTTCTC
LCav3 +25c 3′2	TAGTGGGCCCAGTATGGAGGTTCTCTCATCTCGGACGACGATGCTTTAGGTCCCTCAC
LCav3 ΔAPRASPE +5P 5′	P-ATAGACTCCATGTCATCGCCACGACGACCC
LCav3 ΔAPRASPE +5P 3′	P-CTGCGGGCTGCTAGACGGTCTCG
LCav3 HA 5P 5′	P-GATCCCTACCCCTACGACGTGCCCGACTACGCCCCC
LCav3 HA 5P 3′	P-GATCGGGGGCGTAGTCGGGCACGTCGTAGGGGTAGG
I–IIL (−8b) pET-22b 5′	AAATGTCATATGTCTGAAACAAAGAAGAGAGAGACAGAG
I–IIL (−8b) pET-22b 3′	GAATCTCTCGAGGGCAGTAGGGTTATAGGATAAAGTGC

### RNA extraction and PCR

mRNA for cDNA analyses was extracted [43] from 50–75% and 100% embryos grouped according to morphological features of egg capsules [44] and the shell length of juvenile and adult snails [45].

*Lymnaea* transcripts were amplified by quantitative RT-PCR (qPCR) with primers designed against LCa_v_1 [46], LCa_v_2 [47], LCa_v_3 and −/+ exons 8b and 25c splice isoforms of LCa_v_3 (**Table 3**). qPCR transcripts were normalized against standards, actin, SDHA and HPRT1. Cycle threshold (CT) values for the HPRT1 gene were found to produce the lowest stability value (i.e. 0.098) using NormFinder software [48], indicating its suitability as a reference gene. Expression levels of genes/isoforms were normalized relative to HPRT1 using the ΔΔCT method [49] where ratio = (E_target gene_)^ΔCTtarget gene^/(E_HPRT1_)^ΔCTHPRT1^. Amplicons ranged from 119 to 145 bp, producing single products, with PCR efficiencies (E) ranging from 86 to 110%, with templates generated with 1∶5 serial dilutions of pooled cDNA as templates (**Table 3**). qPCR reactions were carried out in quadruplicate, and standardized between 96 well plate samples with primers against HPRT1.

**Table 3:** Primers used in quantitative RT-PCR experiment (Figure3).

	Real time RT primers:		Ampl. Length	length	Tm (NN)	GC%	qPCR E	R2	Slope
1	Lymnaea actin 5′	CTCACCGACTACCTGATGAAGAT	102	23	55.16	48	**92.9**	**0.998**	**−3.504**
	Lymnaea actin 3′	GTAGCAGAGCTTCTCCTTGATGTC		24	57.48	50			
2	Lymnaea HPRT1 5′	TGTAGAAGACATCATTGACACTGG	145	24	53.86	42	**90.4**	**0.985**	**−3.576**
	Lymnaea HPRT1 3′	GCCAATATAATCTGGTGCGTAAC		23	53.06	43			
3	Lymnaea SDHA 5′	GCTTCAAAGCTGCCTGTATAACTA	122	24	55.74	42	**93.9**	**0.997**	**−3.478**
	Lymnaea SDHA 3′	ATAGAAGTGGTACTGCCAGTGGT		23	55.07	48			
4	LCav1 5′	CCTCATCATCATTGGCTCATT	119	21	51.41	43	**99.1**	**0.990**	**−3.347**
	LCav1 3′	TCTCTCTCAGTTTCTGGAAGTCAC		24	55.56	46			
5	LCav2 5′	TCTCGATGAATATGTTAGGGTCTG	142	24	54.1	42	**107.9**	**0.982**	**−3.147**
	LCav2 3′	GTAGGCCAACTTGTAAGGACACTT		24	54.86	46			
13	LCav3 Universal 5′	CAGAGTGACACAGATGTGCTACAG	139	24	57.34	50	**104.7**	**0.986**	**−3.215**
	LCav3 Universal 3′	GGTTATAGGATAAAGTGCCATGCT		24	54.14	42			
14	LCav3 +8b 5′	AATCCTGAGAAATCTTTGTCCTTG	123	24	53.09	38	**92.5**	**0.954**	**−3.515**
	LCav3 +8b 3′	AGGAGGCTGATGAGATAATGAAAG		24	55.14	42			
15	LCav3 −8b 5′	AGGAAAAAGTCTAAAAAGAAAGGGCT	114	26	55.36	35	**98.6**	**0.995**	**−3.357**
	LCav3 −8b 3′	CTGTAGCACATCTGTGTCACTCTG		24	57.34	50			
16	LCav3 +25c 5′	GGGAGTCTCAAGAAATTGAGGAG	122	23	54.2	48	**95.8**	**0.986**	**−3.426**
	LCav3 +25c 3′	GTATGGAGGTTCTCTCATCTCAGAC		25	55.84	48			
17	LCav3 −25c 5′	GGTTGTAGTTGAAAACTTCCACAAATG	121	27	54.59	37	**91.5**	**0.982**	**−3.545**
	LCav3 −25c 3′	TGGAGGTTCTCTCATCTTCTTTCT		24	55.14	42			
22	LNALCN Universal 5′	GCTTTTACTGGTCCTAATTGATGC	141	24	53.87	42	**100.6**	**0.985**	**3.307**
	LNALCN Universal 3′	AACAGGGCTTCAAGAGAAAATAGA		24	54,19	38			

### Cloning, transfection and electrophysiological recording

Previously, the full-length coding sequence of LCa_v_3 +8b −25c was cloned into the pIRES2-EGFP vector for heterologous expression in HEK-293T cells with bicistronic expression of eGFP (BD Biosciences Clontech) [3]. The other three splice variant combinations, −8b −25c, −8b +25c and +8b +25c as well as +8b (ΔAPRASPE) −25c were created by PCR cassette mutagenesis and reinserted into pIRES2-EGFP vector (**Table 2**).

We use an optimized cell culture and CaPO_4_ transfection strategy for heterologous expression and patch clamp recording of ion channel cDNAs in HEK-293T cells, previously outlined in video protocol format http://www.jove.com/video/2314 [50]. Whole cell patches were sealed with pipette resistances of 2–5 MΩ, and with typical access resistance maintained after breakthrough between 4 and 6 MΩ [3], [50]. Series resistance was compensated to 70% (prediction and correction; 10-µs time lag). Only recordings with minimal leak (<10% of peak) and small current sizes (<500 pA) were used for generating peak current-voltage relationship curves due to loss of voltage clamp above 500 pA; all other recordings were typically maintained below 1.5 nA. Offline leak subtraction was carried out using the Clampfit 10.1 software (Molecular Devices). Voltage-command protocols and the curve fitting of data are described previously [3], [50].

### Measurement of membrane expression

Current densities (pA/pF) were measured from transfections of 6 µg of plasmid of the LCa_v_3 splice variants [50], quantified to equal amounts by plasmid linearization, electrophoresis and densinometric analysis.

### Luminometry

Single haemagglutinin (HA) epitope tags were introduced into the Domain I s5–s6 extracellular loops of the four full-length LCa_v_3 variant constructs. Equimolar amounts of mock, epitope-tagged and un-tagged LCa_v_3 were transfected into HEK-293T cells, and after incubation cells were labeled with rat α-HA monoclonal antibody under membrane permeabilized and non-permeabilized conditions, and luminometry quantified using a FilterMax F5 Multi-Mode Microplate Reader (Molecular Devices) with a chemiluminescence reaction catalyzed with a goat α-rat HRP-conjugated secondary antibody.

### Biotinylation

Surface-expressed channels were measured by biotinylation and identified in Western blots with snail Ca_v_3 channel-specific polyclonal antibodies raised in rabbits using as antigen a large portion of the LCa_v_3 I–II linker peptide lacking exon 8b expressed and purified from bacteria using a pET-22b(+) protein expression vector (**Table 2**). Antibodies were pre-tested for immune reactivity in Western blotting and immunolabeling, as reported previously for antibodies produced strictly against the exon 8b peptide sequence [3]. HEK-293T cell were transfected with equimolar amounts of LCa_v_3 channel variants and biotinylated with Sulfo-NHS-SS-Biotin (Pierce). Surface-labelled, biotinyated fractions were isolated using NeurAvidin® Agarose columns, with the Ca_v_3 channel surface and total protein pools quantified on Western blots using HRP-activated chemiluminescence from a goat α-rabbit secondary antibody.

## Supporting Information

Figure S1 **Genomic region spanning region surrounding tandem 5′ donor splice sites, that lead to the optional inclusion of exon 8b and a conserved APRASPE motif in the I–II linker of snail LCa_v_3 and rat Ca_v_3.1 T-type channels.**
(TIF)

Figure S2 **Genomic region spanning region surrounding tandem 5′ donor splice sites, that lead to the optional inclusion of exon 25c in the III–IV linker of** ***Lymnaea*** **snail LCa_v_3 and other snail T-type channels.**
(TIF)

Figure S3 **Genomic region spanning region surrounding tandem 5′ donor splice sites, that lead to the optional inclusion of exon 25c in the III–IV linker of human T-type channels.** Also shown are the splice sites for exon 26.
(TIF)

Figure S4 **Alignment of amino acid sequences illustrating the conservation of ΔΔ and 25c alternative splice isoforms in the III–IV linker of snail LCa_v_3 and vertebrate Ca_v_3.1 and Ca_v_3.2 channels.** Illustration of the aligned (A) amino acid sequences and (B) DNA sequences flanking exons ΔΔ, 25c and 26. Presence of Exon 25c creates a consensus protein kinase A site (boxed) in LCa_v_3, Ca_v_3.1 and Ca_v_3.2 channels. Optional Exon 26 is only found in Ca_v_3.1 and Ca_v_3.2 channels. Ca_v_3.3 only has the ΔΔ exon isoform. Intron sizes are in brackets.
(TIF)

Figure S5 **PCR amplification of adult and embryonic cDNAs spanning the III–IV linker coding sequence of snail LCa_v_3 reveal two mRNA transcripts coding for ΔΔ and 25c alternative-splice isoforms, but not optional cassette exon 26.** (A) Original PCR of III–IV linker inserts derived from adult and embryonic mRNA from snails. (B) High resolution and quantification of the two III–IV linker gel insert sizes using 1 kb DNA Lab-On-Chip technology with Experion (Bio-Rad) automated gel electrophoresis system. (C) Diagnostic gel pattern of the two different clone sizes in cloned pGEM-T Easy insert samples, assayed by *EcoR*I restriction digest and confirmed by DNA sequencing. Note the predominance of ΔΔ clones over exon 25c in samples of embryonic RT-PCR clones.
(TIF)

Figure S6 **Scatter matrix analysis of normalized mRNA qPCR values for the various alternative splice sites of LCa_v_3 reveals that +8b and −25c variants tend to have similar expression patterns amongst the various adult and juvenile tissues tested (correlation coefficient R^2^ of 0.885); −8b and +25c also have a high R^2^ value of 0.898.** Confidence regions of 95% for the correlated values is depicted by the red ellipses. Analysis was carried out using Origin 8.5 software (OriginLab).
(TIF)

Figure S7 **Confirmation of specificity of snail LCav3 polyclonal antibodies for bacterially-expressed epitope peptide using Western blotting.** Polyclonal antibodies raised against a 17.6 kDa peptide corresponding to the I–II linker of LCav3 lacking exon 8b, detect bacteria-expressed and Histidine tag-purified I–II linker protein on western blots (white arrow), and do not detect a similarly expressed 23 kDa protein corresponding to exon 8b (black arrow).
(TIF)

Figure S8 **Membrane-expression of HA-tagged LCav3 variants could not be measured using luminometry.** More than a doubling of ELISA signal was recorded when permeabilized transfected cells were compared to non-permeabilized conditions, regardless of the treatment, including cells transfected with HA-epitope tags or untagged (wt) LCa_v_3 channels. HA epitopes were introduced into the IS5–S6 extracellular loops of the LCav3 variants, and identified in transfected homogenates of HEK-293T cells with labelled anti-rat HA monoclonal antibody. Secondary goat anti-rat HRP (horse radish peroxidase) catalyzed the chemilluminescence quantified using a FilterMax F5 Multi-Mode Microplate Reader (Molecular Devices).
(TIF)

Figure S9 **HA-tagged LCav3 variants expressed >20 fold less than un-tagged LCav3 variants in HEK-293T cells.** Above is the largest-sized currents of three LCav3 variants (+8b−25c, −8b−25c, and +8b+25c) transfected and recorded in HEK-239T cells by whole cell patch clamp technique and generated by voltage-steps to −40 mV from −110 mV holding potential. These currents for HA-tagged channels were carried out under optimal transfection efficiency and culturing conditions. Usually for untagged clones, peak currents were usually greater than 2000 pA, recorded three three days post-transfection, and we selected for smaller currents. No recorded HA-tagged channel was as large as 100 pA.
(TIF)

Methods S1 **Supplementary Materials and Methods.**
(DOCX)
