# Correction: Gene Transcription and Splicing of T-Type Channels Are Evolutionarily-Conserved Strategies for Regulating Channel Expression and Gating

**DOI:** 10.1371/annotation/9d69fd63-4378-4e05-b2ad-2a5c98561223

**Published:** 2013-09-19

**Authors:** Adriano Senatore, J. David Spafford

The following information was missing from the Funding section: Heart and Stroke Foundation of Canada Grant. 

