# Supplementary figures and images for: Gene Transcription and Splicing of T-Type Channels Are Evolutionarily-Conserved Strategies for Regulating Channel Expression and Gating
Source: PLoS One. 2012 Jun 15;7(6):e37409. doi: 10.1371/journal.pone.0037409 (PMC3376122; doi:10.1371/journal.pone.0037409)

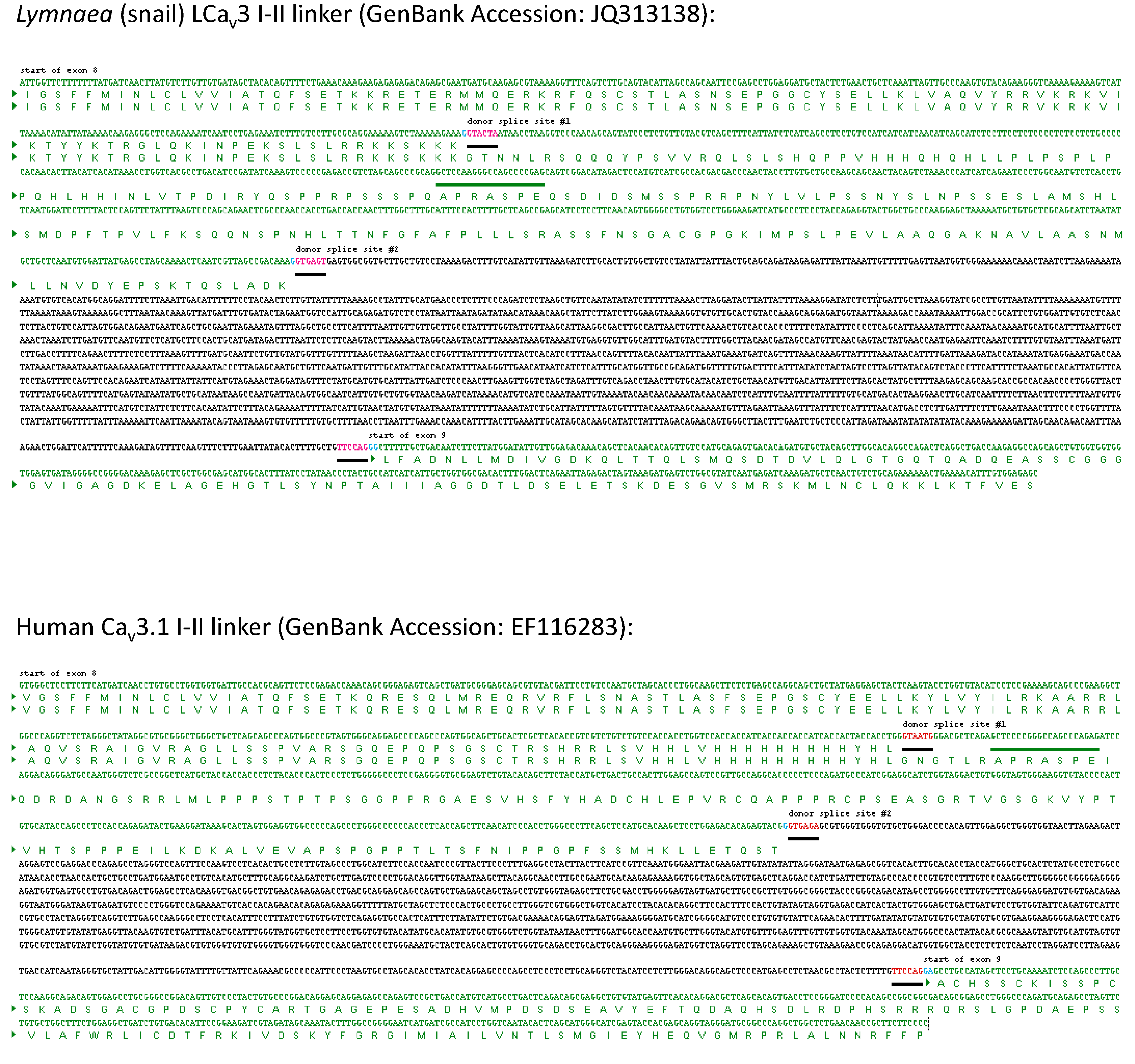

Supplement: Figure S1 — Genomic region spanning region surrounding tandem 5′ donor splice sites, that lead to the optional inclusion of exon 8b and a conserved APRASPE motif in the I–II linker of snail LCav3 and rat Cav3.1 T-type channels. (TIF) [file pone.0037409.s001.tif]

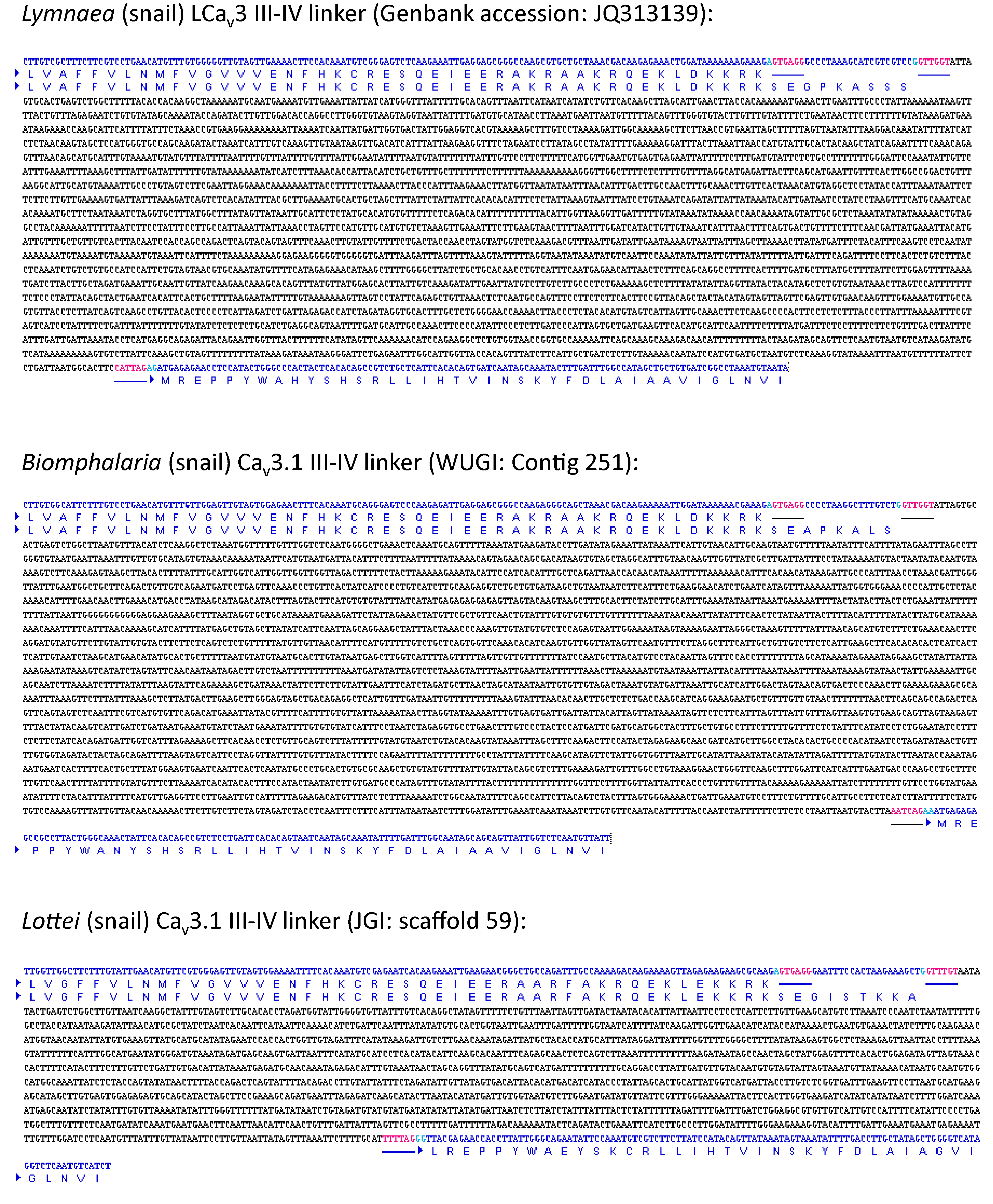

Supplement: Figure S2 — Genomic region spanning region surrounding tandem 5′ donor splice sites, that lead to the optional inclusion of exon 25c in the III–IV linker of Lymnaea snail LCav3 and other snail T-type channels. (TIF) [file pone.0037409.s002.tif]

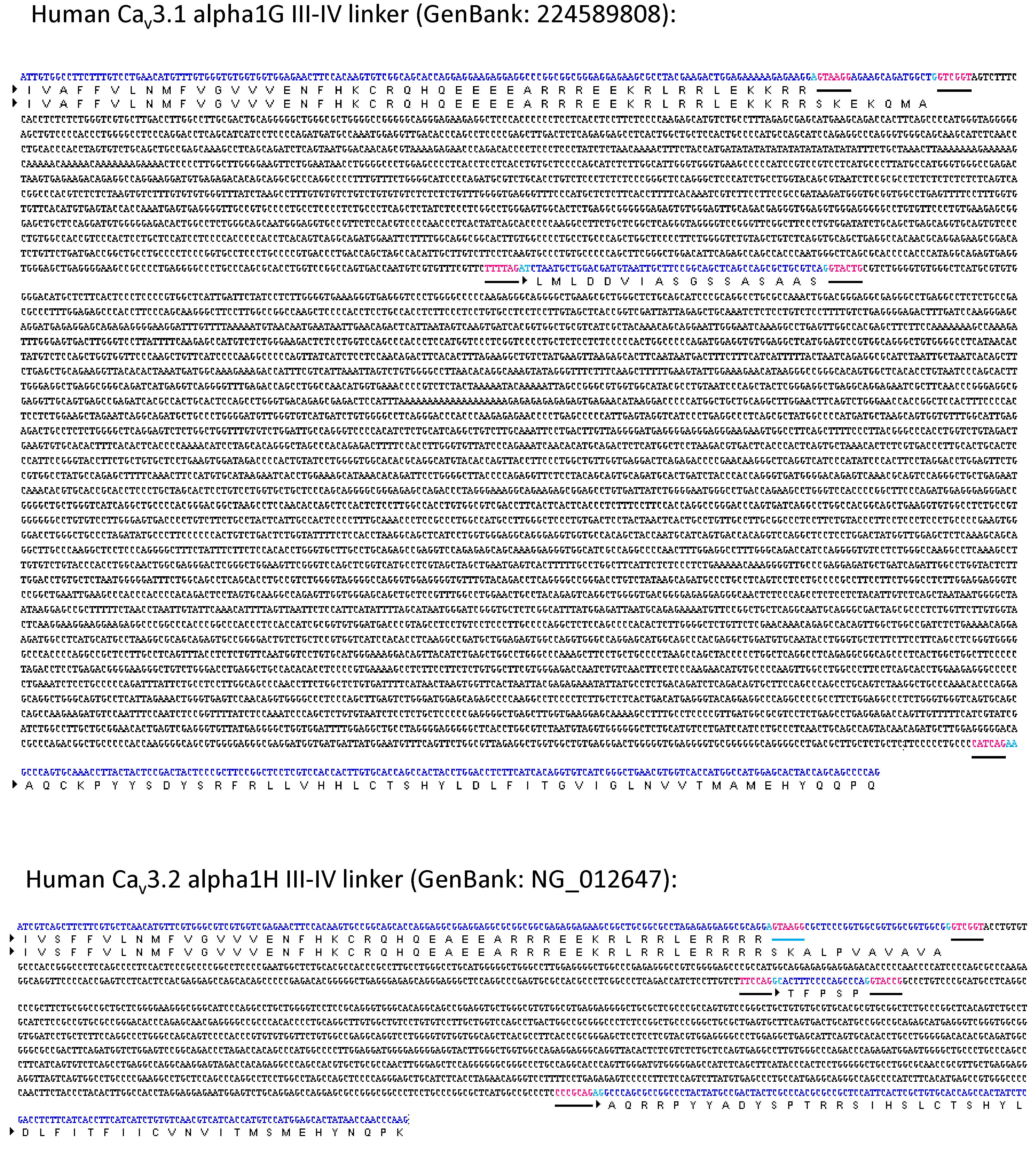

Supplement: Figure S3 — Genomic region spanning region surrounding tandem 5′ donor splice sites, that lead to the optional inclusion of exon 25c in the III–IV linker of human T-type channels. Also shown are the splice sites for exon 26. (TIF) [file pone.0037409.s003.tif]

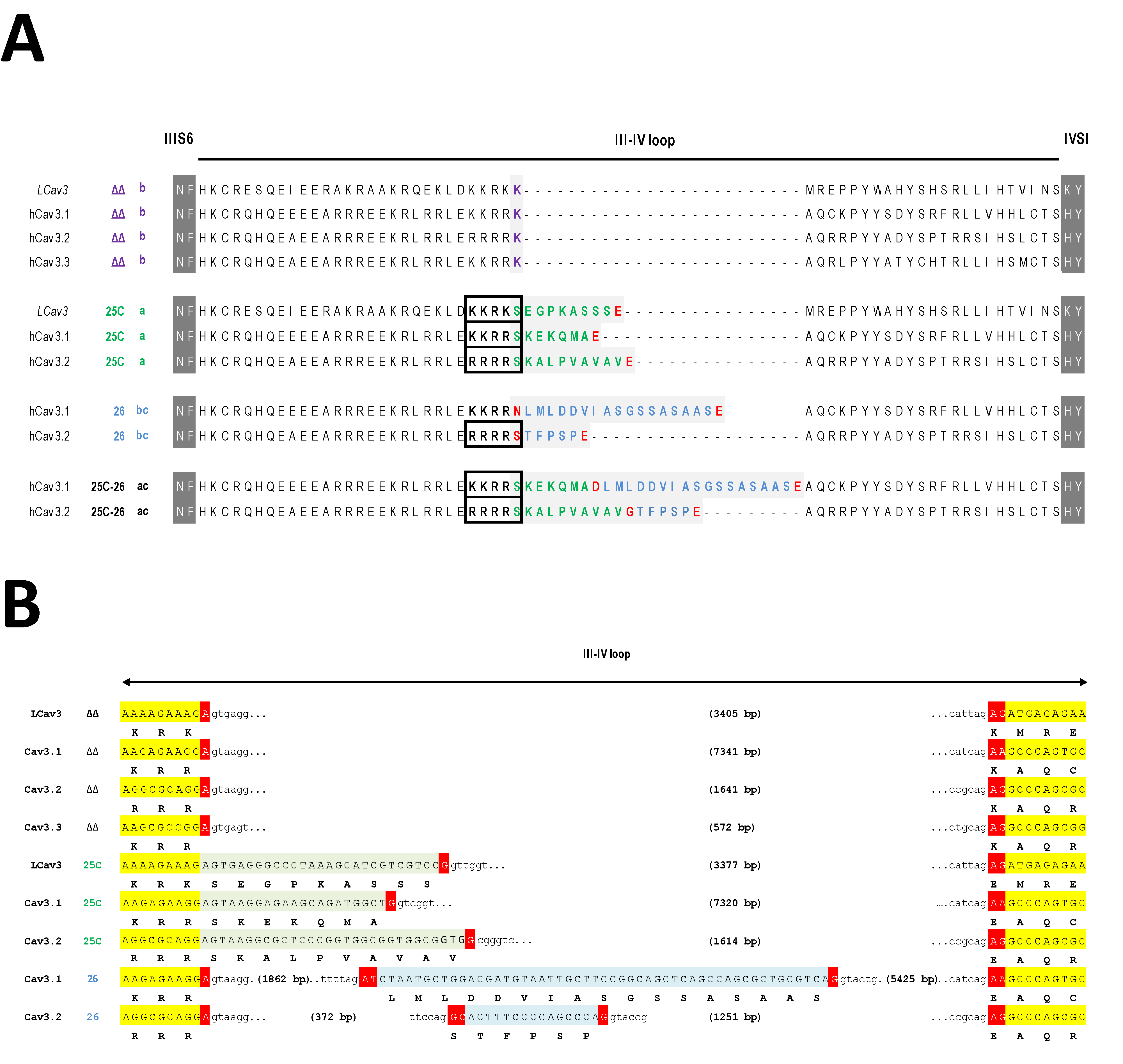

Supplement: Figure S4 — Alignment of amino acid sequences illustrating the conservation of ΔΔ and 25c alternative splice isoforms in the III–IV linker of snail LCav3 and vertebrate Cav3.1 and Cav3.2 channels. Illustration of the aligned (A) amino acid sequences and (B) DNA sequences flanking exons ΔΔ, 25c and 26. Presence of Exon 25c creates a consensus protein kinase A site (boxed) in LCav3, Cav3.1 and Cav3.2 channels. Optional Exon 26 is only found in Cav3.1 and Cav3.2 channels. Cav3.3 only has the ΔΔ exon isoform. Intron sizes are in brackets. (TIF) [file pone.0037409.s004.tif]

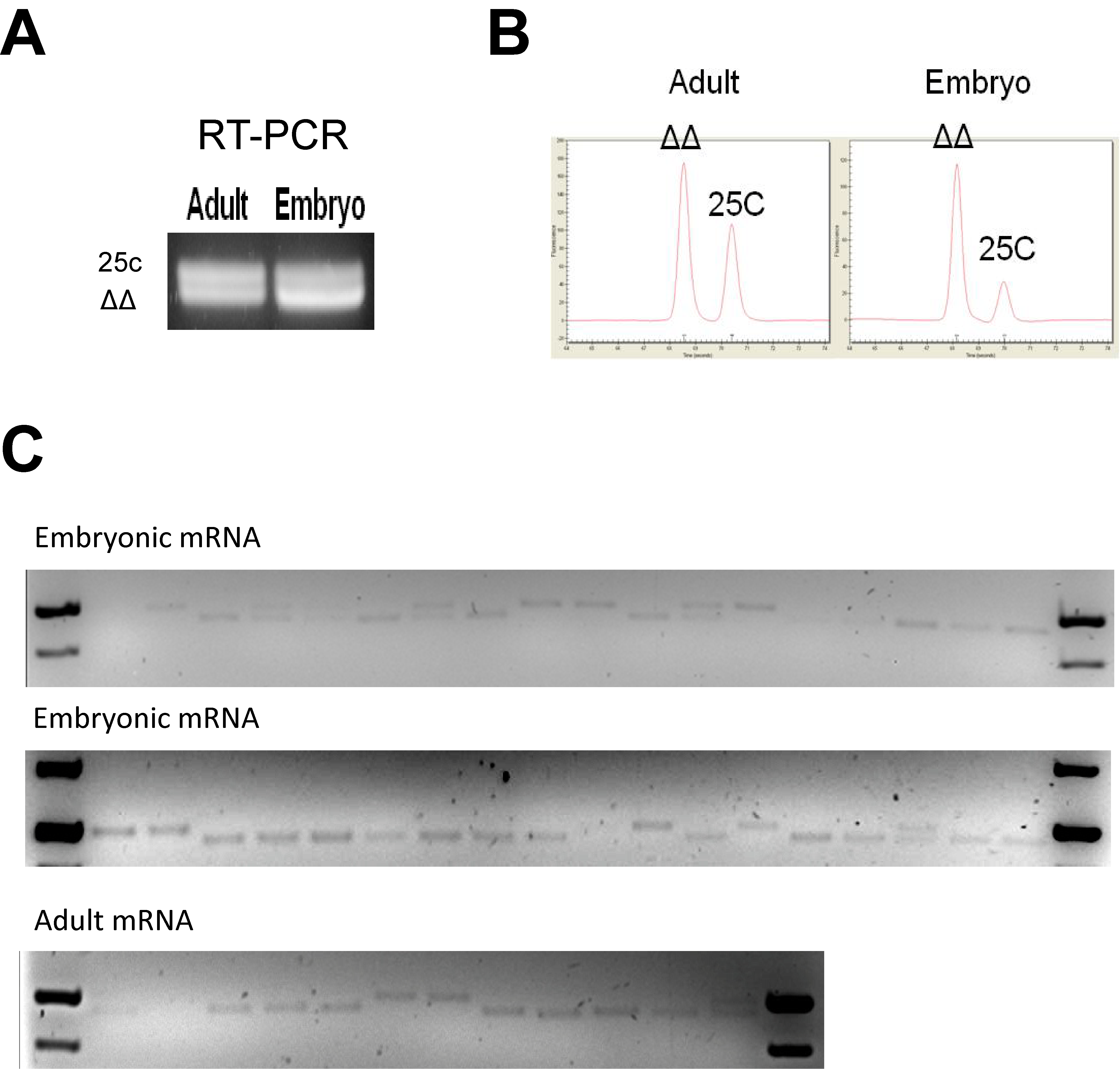

Supplement: Figure S5 — PCR amplification of adult and embryonic cDNAs spanning the III–IV linker coding sequence of snail LCav3 reveal two mRNA transcripts coding for ΔΔ and 25c alternative-splice isoforms, but not optional cassette exon 26. (A) Original PCR of III–IV linker inserts derived from adult and embryonic mRNA from snails. (B) High resolution and quantification of the two III–IV linker gel insert sizes using 1 kb DNA Lab-On-Chip technology with Experion (Bio-Rad) automated gel electrophoresis system. (C) Diagnostic gel pattern of the two different clone sizes in cloned pGEM-T Easy insert samples, assayed by EcoRI restriction digest and confirmed by DNA sequencing. Note the predominance of ΔΔ clones over exon 25c in samples of embryonic RT-PCR clones. (TIF) [file pone.0037409.s005.tif]

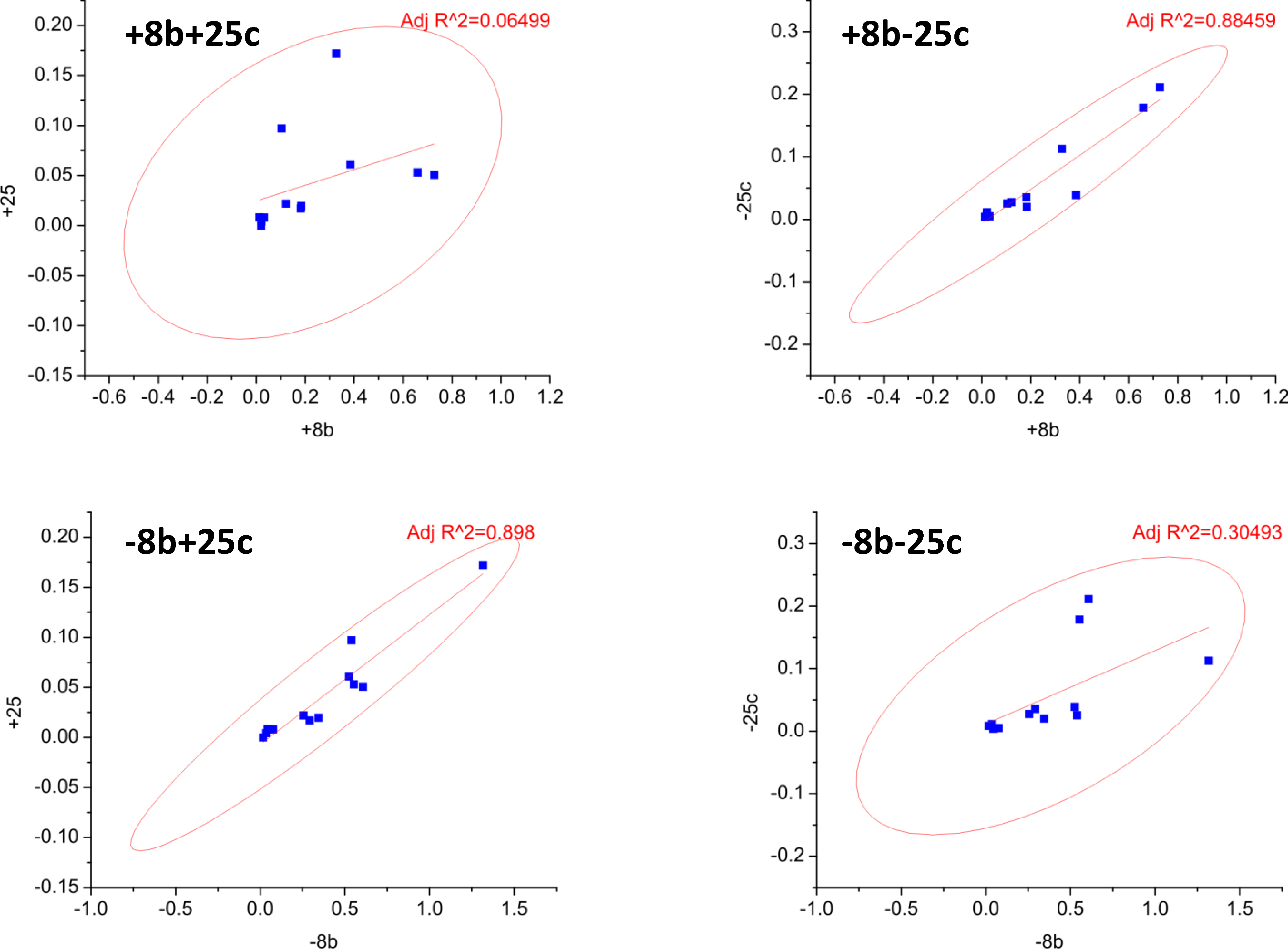

Supplement: Figure S6 — Scatter matrix analysis of normalized mRNA qPCR values for the various alternative splice sites of LCav3 reveals that +8b and −25c variants tend to have similar expression patterns amongst the various adult and juvenile tissues tested (correlation coefficient R2 of 0.885); −8b and +25c also have a high R2 value of 0.898. Confidence regions of 95% for the correlated values is depicted by the red ellipses. Analysis was carried out using Origin 8.5 software (OriginLab). (TIF) [file pone.0037409.s006.tif]

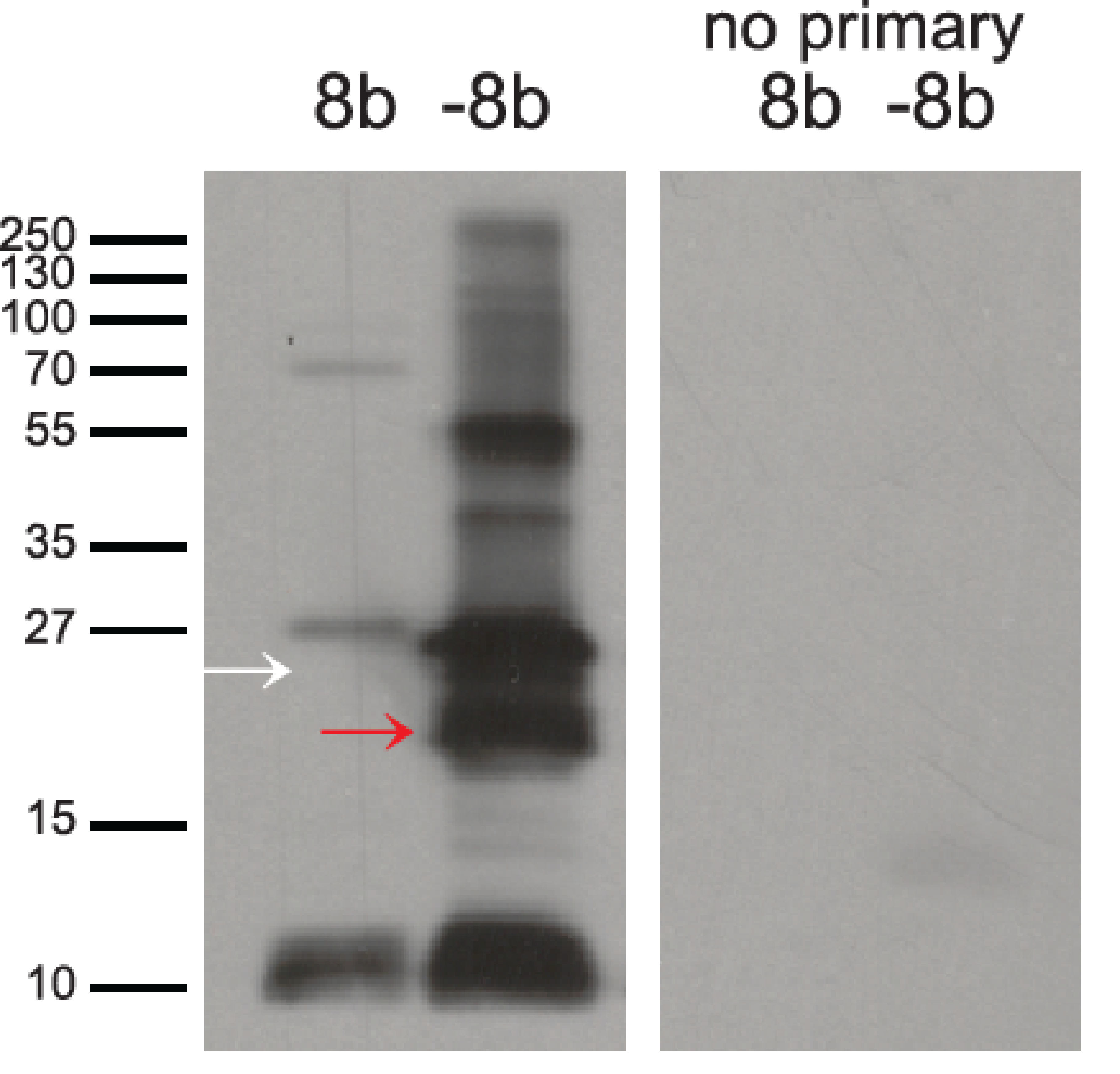

Supplement: Figure S7 — Confirmation of specificity of snail LCav3 polyclonal antibodies for bacterially-expressed epitope peptide using Western blotting. Polyclonal antibodies raised against a 17.6 kDa peptide corresponding to the I–II linker of LCav3 lacking exon 8b, detect bacteria-expressed and Histidine tag-purified I–II linker protein on western blots (white arrow), and do not detect a similarly expressed 23 kDa protein corresponding to exon 8b (black arrow). (TIF) [file pone.0037409.s007.tif]

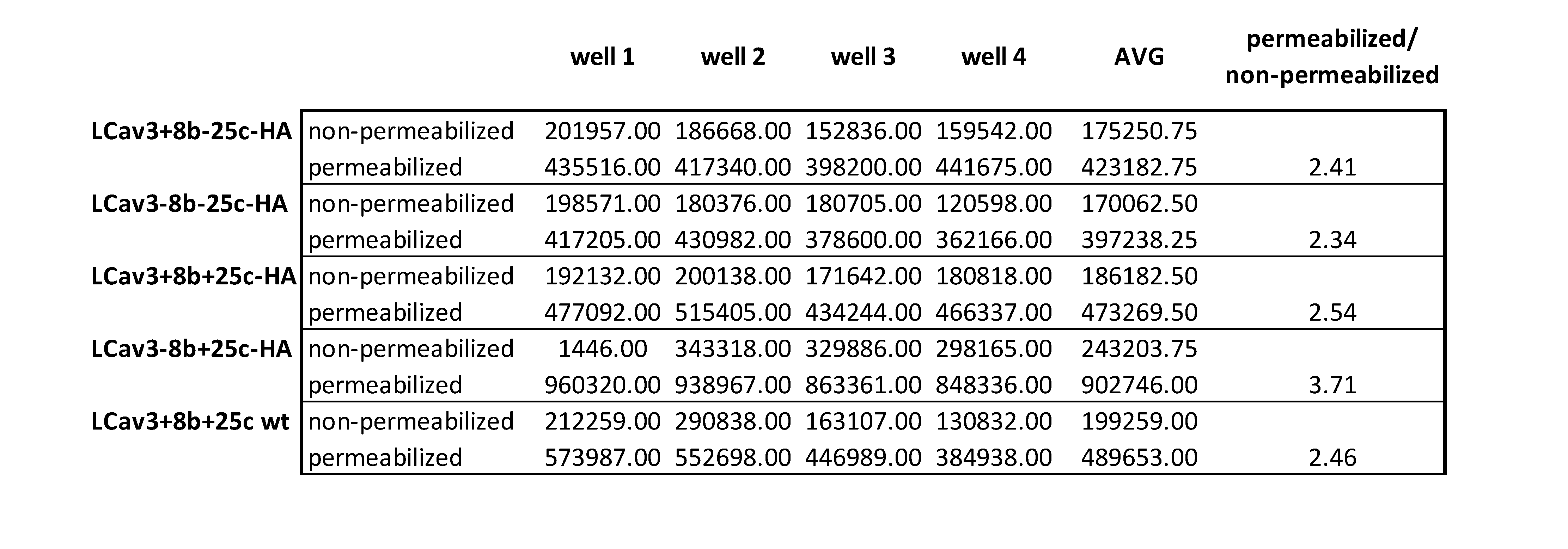

Supplement: Figure S8 — Membrane-expression of HA-tagged LCav3 variants could not be measured using luminometry. More than a doubling of ELISA signal was recorded when permeabilized transfected cells were compared to non-permeabilized conditions, regardless of the treatment, including cells transfected with HA-epitope tags or untagged (wt) LCav3 channels. HA epitopes were introduced into the IS5–S6 extracellular loops of the LCav3 variants, and identified in transfected homogenates of HEK-293T cells with labelled anti-rat HA monoclonal antibody. Secondary goat anti-rat HRP (horse radish peroxidase) catalyzed the chemilluminescence quantified using a FilterMax F5 Multi-Mode Microplate Reader (Molecular Devices). (TIF) [file pone.0037409.s008.tif]
