# Supplementary material for: Gene Transcription and Splicing of T-Type Channels Are Evolutionarily-Conserved Strategies for Regulating Channel Expression and Gating
Source: PLoS One. 2012 Jun 15;7(6):e37409. doi: 10.1371/journal.pone.0037409 (PMC3376122; doi:10.1371/journal.pone.0037409)
Supplement: Methods S1 — Supplementary Materials and Methods. (DOCX) [file pone.0037409.s010.docx]

**Supplementary Materials and Methods**:

**Identification of LCa_v_3 channel splice variants.** During the initial sequencing and cloning of LCa_v_3 (1), multiple sites containing splice variability were identified, including conserved optional exons 8b in the I-II linker and 25c in the III-IV linker. Exon 26, found in the III-IV linkers of mammalian Ca_v_3.1 and Ca_v_3.2 channel genes was not detected in these preliminary studies, prompting us to do a comprehensive search for an equivalent exon in LCa_v_3. Briefly, *Lymnaea* adult CNS and embryonic whole animal cDNAs were PCR-screened using two nested primer pairs flanking the variable site of the III-IV linker coding sequence (**Table 2**). Only two DNA bands were visible in ethidium bromide-stained agarose gels after electrophoresis (**Figure S5**), whose intensity suggested an enrichment of the ∆∆ variant in the embryo. Since exon 25c and 26 PCR products could be of similar size, secondary PCRs were repeated and further analyzed using an Experion^TM^ automated DNA 1K electrophoresis system (BioRad), which provides both high resolution of separated DNA molecules and quantitative output of their relative abundance. Only two sizes were detectable, and the corresponding peak signal amplitudes demonstrate that the ∆∆ variant is enriched in the embryo (**Figure S5**). These same DNA products were gel-purified and ligated into pGEM-T Easy Vector (Promega), the ligations were transformed into *E. coli* DH5α, and the resulting white colonies were used for plasmid isolation via the alkaline lysis method (modified from Bimboim and Doly 1979 (2). Plasmids were then restriction digested with *EcoR*I (NEB), which flanks the multiple cloning site of the pGEM-T Easy, and the sizes of cloned PCR amplified inserts were evaluated using agarose gel electrophoresis. Again only two sizes were visible (**Figure S5**), and sequencing of several clones of either size failed to identify an exon 26.

To assess whether the splicing of LCa_v_3 transcripts in the I-II and III-IV linker coding sequences resembles that of Ca_v_3.1 and Ca_v_3.2, we sequenced the genomic DNA corresponding to these regions along the gene. Genomic DNA was isolated from adult animals ground in liquid nitrogen (3), and this was used as template for nested PCRs with primers flanking respective intron splice sites (**Table 2**). PCR reactions were carried out using high fidelity DNA polymerase PfuTurbo AD (Agilent Technologies Inc.), and amplified DNA was electrophoresed, gel purified, and subject to multiple rounds of sequencing to obtain the complete sequence (**Figure S1, Figure S2**).

**Measuring mRNA expression levels of LCav3 channel splice variants**

**Tissue isolation and RNA extraction.** The embryonic development schedule was adopted from Marois and Croll, 1992 (4) and Nagy and Elekes, 2002 (5) and spans between a range of 1 to 100% development based on morphological and histological criteria. The juvenile to adult development schedule was adopted from McComb, Varshney and Luckowiak, 2005 (6) and is based on shell length. Embr­yonic development was assessed by observation of egg capsules under a light microscope, and sacs were grouped into two groups of 50-75% and 100% developed. Juveniles were selected and grouped based on shell lengths of 1.0 to 1.5 cm while adults contained shells 2.0 to 2.5 cm in length; this criteria was previously shown to separate non-sexually mature juvenile animals from sexually mature adults. For whole animal RNA extracts, egg capsules or entire animals were ground in liquid nitrogen and stored in 1.5 mL centrifuge tubes at -80^o^C until RNA extraction. Juvenile and adult organs were dissected from anesthetised animals (10% v/v Listerine 5 to 10 min) and placed directly into 1.5 mL tubes in liquid nitrogen. RNA was extracted with Tri-Reagent (7), quantified using UV spectrophotometry and quality assessed by agarose gel electrophoresis and visualization of ethidium bromide-stained gels under UV light. For DNase treatment, 10 µg of each RNA extract was diluted to 100 ng/µL in a volume of 100 µL in separate 1.5 mL tubes and to each added 12 µL of 10x DNase buffer (Ambion), 2 µL of RiboLockTM RNase inhibitor (Fermentas) and 2 µL of DNAse I enzyme (Ambion) and tubes were incubated at 37^o^C for 30 minutes. Samples were then treated with an equal volume of 50:50 v/v phenol/chloroform, precipitated with ethanol, and resuspended with 50 µL DEPC-treated water.

**Reverse transcription and qPCR.** Reverse transcription was carried out using a iScriptTM Reverse Transcriptase Supermix (BioRad) with each reaction containing 800 ng of RNA, 5 µL of iScript RT supermix, and DEPC water for a total of 20 µL. Temperature cycling for the reactions was 25^o^C for 5 min, 42^o^C for 30 min, then 85^o^C for 5 min. qPCR primers were designed to selectively amplify a universal region and specific splice variants of *Lymnaea* LCa_v_3 transcripts, and universal primers were designed for LCa_v_3 as well as Lymnaea neuronal α_2_δ, LCa_v_1, and LCa_v_2 (**Table 3**). The specificity of PCR primers for particular splice isoforms was assessed by comparing the size of PCR products amplified from a pooled qPCR cDNA library with those amplified from cloned cDNAs, as well as by analysing the melting curves of PCR products after all qPCR experiments. Amplicons ranged from 119 to 145 bp, and PCR primer efficiency was determined using relative standard curves generated with 1:5 serial dilutions of pooled cDNA as templates. All primers were found to have PCR efficiencies (E) ranging from 86 to 110%. qPCR reactions were done in quadruplicate 10 µL reactions using, each containing 5 µL of SsoFastTM EvaGreen® Supermix, 0.5 µL of each primer at an initial concentration of 10 µM, 3 µL of water, and 1 µL of template cDNA. For negative controls, used 1 µL of DNase-treated RNA at 40 ng/µL instead of cDNA, and 1 µL of DEPC water for no the template controls. To standardize between plates, each contained triplicate qPCR reactions using primers against HPRT1 (see below) and 1:5 diluted pooled qPCR cDNA as template. All cycle thresholds (CT) used for analysis were determined relative to the average CT of the HPRT1 controls in each plate. PCR amplification and fluorescence reading was done in a Bio-Rad C1000TM Thermal Cycler equipped with a CFX96TM Real-Time System, with cycling parameters of 90^o^C for 30 seconds followed by 40 cycles of 95^o^C for 5 seconds and 56^o^C for 5 seconds.

**qPCR data analysis.** Primers were designed against *Lymnaea* actin (GenBank accession number DQ206431), subunit A of the succinate dehydrogenase complex (SDHA; GenBank accession number ES578163.1), and hypoxanthine phosphoribosyltransferase 1 (HPRT1; GenBank accession number ES571571.1) for use as reference genes (**Table 3**). CT values for the HPRT1 gene were found to produce the lowest stability value (i.e. 0.098) using NormFinder software (8), indicating its suitability as a reference gene. Conversely, actin and less so SDHA CT values varied considerably between tissues (stability values of 0.634 and 0.296, respectively). To quantify relative expression levels of genes/splice isoforms of interest relative to HPRT1, data was analyzed using the ∆∆CT method (9) where ratio = (E_target gene_)^∆CTtarget gene^/(E_HPRT1_)^∆CTHPRT1^.

**Cloning of LCa_v_3 channel splice variants for expression in HEK-293T cells.** Previously, the full-length coding sequence of LCa_v_3 +8c -25c was cloned into the pIRES2-EGFP vector for heterologous expression in HEK-293T cells with bicistronic expression of eGFP (BD Biosciences Clontech) (1). To create the -8b sub-clone, a *Bgl*II to *Sal*I fragment was excised from the full length clone (containing exon 8b) and inserted into a circularized pGEM-T Easy vector (Promega) that had been modified by insertion of annealed oligonucelotides (**Table 2**) into *Nco*I of the vector to introduce a *Bgl*II site. Removal of the exon 8b coding sequence was achieved by doing PCR (with high fidelity PfuTurbo AD DNA polymerase), using the entire subclone as template and primers flanking exon 8b whose 3’ ends were directed away from the optional exon such that PCR amplification would produce a linear plasmid lacking 8b. 5’ phosphate groups on the primers enabled blunt-end ligation and re-circularization of the PCR product, which was then transformed into Stbl2 bacteria (Invitrogen) for plasmid isolation. After confirmation of sequence, the -8b *Bgl*II to *Sal*I DNA fragment was re-inserted into the full-length LCa_v_3 clone to create a full-length -8b -25c variant. To create the +25c sub-clone, a *Sal*I to *Mlu*I fragment of the original full-length LCa_v_3 clone was inserted into circularized pGEM-T Easy. Two rounds of Assembly PCR were used with the -25c sub-clone as template with 3’ primers designed to introduce the exon 25c coding sequence followed by an *Apa*I restriction site present in LCa_v_3 just downstream of 25c (**Table 2**). The PCR product containing the additional 25c sequence was then cloned back into the *Mlu*I-*Sal*I sub-clone via *Apa*I, present in both the vector and the LCa_v_3 coding sequence, and confirmed by sequencing. The modified *Sal*I to *Mlu*I cDNA was then cloned back into the full-length LCa_v_3 +8b -25c and -8b -25c pIRES2-EGFP clones to create LCa_v_3 +8b +25c and LCa_v_3 -8b +25c in pIRES2-EGFP, respectively. The ∆APRASPE sub-clone was created in a similar manner as -8b, with primers that removed the coding sequence for APRASPEQSD (**Table 2**). The mutant *Bgl*II-*Sal*I DNA was cloned back into the full-length LCa_v_3 +8b -25c to create Lca_v_3 +8b(∆APRASPE) -25c in pIRES2-EGFP.

**Transfection and electrophysiological recording.** Previously, we reported an optimized cell culture and CaPO_4_ transfection strategy for heterologous expression of ion channel cDNAs in HEK-293T cells for electrophysiological recording (10). This methodology was used for all transfections of LCa_v_3 splice variant vectors for electrophysiological recording, using 6 µg of vector DNA per transfection. Whole-cell patch clamp technique was done as previously reported (1, 10) using a 2 mM external calcium solution (2 mM CaCl_2_, 160 mM TEA-Cl, 10 mM HEPES pH 7.4 with TEA-OH) and an internal solution containing 110 mM CsCl, 10 mM EGTA, 3 mM Mg-ATP, 0.6 mM Na-GTP, and 10 mM HEPES pH 7.2 with CsOH (11). All chemicals were from SIGMA. Recordings were done at room temperature; the pipette resistance was maintained between 2 and 5 megaohms, and the typical access resistance between 4 and 6 megaohms. Series resistance was compensated to 70% (prediction and correction; 10-μs time lag). Only recordings with minimal leak (<10% of peak) and small current sizes (<500 pA) were used for generating peak current-voltage relationship curves due to the loss of voltage clamp above 500 pA; all other recordings were typically maintained below 1.5 nA with the exception of current density analysis (see below). Offline leak subtraction was carried out using the Clampfit 10.1 software (Molecular Devices).

**Data Analysis of electrophysiological data.** Summary of electrophysiology data is provided in **Table 1**. For the calcium current activation plots corresponding to cloned LCa_v_3 channel variants, the peak current values from each current-voltage relationship data set were converted to conductance values using the equation gCa = I_peak_/(V_command_ – E_Ca_), where gCa is the calcium conductance, I_peak_ is the peak calcium current, V_command_ is the command potential, and E_Ca_ is the Ca^2+^ reversal potential extrapolated from the linear ascending portion of the averaged current-voltage relationship curve for each variant. Conductance values were then normalized and individually fitted with the Boltzmann equation, g/g_max_ = (1 + (exp(−V_command_ – V_½_)/K)) – 1, where g is the peak Ca^2+^ conductance, g_max_ is the maximal peak conductance, V_command_ is the conditioning potential, V_½_ is the half-maximal activation, and k is the activation slope factor. Steady-state inactivation curves were constructed by plotting normalized peak currents (peak test pulse current/peak pre-pulse current) as a function of various inactivating potentials. The data were fitted with a Boltzmann equation, I/I_max_ = (1 + exp((V_inact_ – V_½_/k)) – 1, where I is the peak test pulse current, I_max_ is the peak test pulse current from a conditioning potential of -110 mV, V_inact_ and V_½_ are the conditioning potential and the half-maximal inactivation, respectively, and k is the inactivation slope factor. Kinetics of activation, inactivation, and deactivation were determined by fitting monoexponential functions over the growing or decaying phases of each current trace using the software Clampfit 10.1. Recovery from inactivation of peak Ca^2+^ currents, elicited by stepping to -35 mV, were assessed for each channel variant using a two pulse protocol, with a 1 second pre-pulse to inactivate channels paired to subsequent test pulses occurring at increasing time intervals from the inactivating pre-pulse to measure recovery. Plots of the standardized recovery (test pulse peak current/pre-pulse peak current) as a function of the time between pre- and test pulses were fitted with monoexponential curve functions.

**Measurement of membrane expression of Lca_v_3 channel splice variants in HEK-293T cells**

**Current density analysis.** Equal amounts of all four LCa_v_3 splice variant and ∆APRASPE pIRES2 contructs (6 µg: quantified by plasmid linearization, electrophoresis and densinometric analysis on ethidium bromide-stained agarose gels) were transfected into HEK-293T cells as previously reported (10). The next day cells were washed and left over night at 28°C in a humidified, 5% CO_2_ chamber. After incubation, cells were detached using a trypsin-EDTA solution (Sigma), plated at ~10% confluency onto glass coverslips, and incubated at 37 °C for 1 h to permit adhesion to the glass substrate (12). For peak current, 250 ms steps from -110 mV to -42.5 mV were taken, and the cell capacitance was recorded. Current densities (pA/pF) were determined by dividing the peak current by the cell capacitance of each cell.

**Luminometry.** Briefly, the coding sequence for the haemagglutinin (HA) epitope was introduced into the domain I s5-s6 extracellular loops of the four full-length LCa_v_3 variant constructs using an annealed oligonucleotide adaptor bearing 5’ phosphates and single-stranded overhangs complementary to a *Bgl*II site in LCa_v_3 (**Table 2**). Agarose gel electrophoresis and ethidium bromide-based quantification of the four HA-tagged LCa_v_3 channel constructs was used to ensure transfection of equimolar amounts plasmid (~8 µg depending on size of constructs) into ~80% confluent HEK-293T cells in 6 mL flasks, each transfection having 18.7 µL of Lipofectamine LTX reagent (Invitrogen) and a total volume of 1.5 mL with OptiMEM (Sigma). For controls, mock-transfected cells and cells transfected with untagged Lca_v_3 (+8b +25c) were included in the experiment. After transfection the cells were incubated at 28^o^C for 2 days, following which cells were split each into 4 wells of poly-L-lysine-coated (Sigma) 24-well plates (Black Visiplate^TM^, PerkinElmer), incubated at 37^o^C for 3 hours, washed twice with warm PBS (37^o^C), then fixed with warm PBS containing 4% PFA for 5 minutes. Cells were then washed 2 x 5 minutes with PBS, and ½ of wells were permeabilized with 0.1% Triton X-100 in PBS for 5 minutes and all wells washed 3 x 5 minutes with PBS. All cells were blocked for 50 min at room temperature (1% FBS, 0.05% TWEEN-20 in PBS), incubated with rat α-HA monoclonal antibody (Roche), washed 4 x 10 minutes (1:1000 in PBS with 1% FBS, 0.05% TWEEN-20), and incubated with goat α-rat HRP-conjugated secondary antibody (1:1000 in PBS + 0.05% TWEEN-20) for 30 minutes. After washing 3 x 10 minutes with PBS, 400 µL of SuperSignal® ELISA Femto Maximum Sensitivity Substrate (Thermo SCIENTIFIC) and plates were detected using a FilterMax F5 Multi-Mode Microplate Reader (Molecular Devices) using the luminometer setting of SoftMax Pro 6.1 software (Molecular Devices) for data acquisition and analysis.

**Biotinylation.** Polyclonal antibodies were produced in rabbits using as antigen a large portion of the LCa_v_3 I-II linker peptide lacking exon 8b. The desired coding sequence was PCR-amplified (**for primers see Table 2**) and cloned into pET-22b(+) bacterial protein expression vector (Novagen) via *Nde*I and *Xho*I restriction sites, and the 17.6 kDa protein was expressed, purified, dialysed, and injected into rabbits as previously reported (1). Notably, during dialysis prior to injection, some of the protein precipitated, however a substantial amount remained in solution. It is expected that the precipitated component might actually have enhanced antigenicity due to more stability of precipitated proteins in the interstitial fluid. IgG rabbit antiserum was tested for immune reactivity with the purified antigen by Western blotting (not shown), and further tested in LCa_v_3-transfected HEK-293T cells, where we performed immunolabeling experiments with cells transfected with either 8 μg of LCa_v_3 +8b -25c in pIRES2-EGFP or with 3.2 μg of LCa_v_1 α1 subunit in pIRES2-EGFP (13) plus 2.4 μg of the rat β1 subunit in pMT2 and 2.4 μg of rat α_2_δ in pMT2 as previously reported (1). AlexaFluor® 594 goat anti-rabbit IgG was used as the secondary antibody (Invitrogen), and cells were imaged at 40 × magnification using a Zeiss AxioObserver Z1 inverted epifluorescent microscope to detect the AlexaFluor® 594 antibody and eGFP. Images were captured using Zeiss AxioVision software, and brightness/contrast was adjusted using Adobe Photoshop.

For biotinylation of surface expressed channels we used the Pierce® Cell Surface Protein Isolation Kit. Briefly, a fully confluent 6 mL flask of HEK-293T cells was split 1:6 into 5 new flasks. After 5 hours at 37^o^C, media was changed to no antibiotic and flasks left over night at 37^o^C. The following day, equimolar amounts of the four LCa_v_3 channel variants were transfected into the cells (~ 6 µg; quantified and transfected as indicated in the current density section of the materials and methods above), and cells were incubated at 37^o^C for 6 hours then media replaced with 6 mL of media lacking antibiotics. The next day, cells were transferred to 28^o^C for overnight and subsequently washed once with 5 mL of warm (37^o^C) PBS and once with 5 mL of ice cold PBS. 3.5 mL of ice-cold Sulfo-NHS-SS-Biotin (Pierce) was then added to each flask and these were incubated on a rocking platform at 4^o^C for 30 minutes. 175 µL of Quenching Solution was added to each flask and the cells were gently resuspended and transferred to separate 5 mL conical tubes; remaining cells were obtained using 830 µL of TBS to rinse out the flasks using a micropipette. Tubes were then centrifuged in a swinging bucket rotor at 500 x g for 3 minutes, and the supernatant was discarded. Cell lysis was achieved using 130 µL of the provided Lysis Buffer, supplemented with 1/10^th^ volume of CALBIOCHEM Protease Inhibitor Cocktail III in 1.5 mL centrifuge tubes, and sonication at low power (e.g. 3.5) on ice using 5-second bursts over the course of 45 minutes. Lysates were centrifuged at 10 000 x g for 2 minutes at 4^o^C, and supernatants transferred to new tubes. 100 µL of each supernatant was removed for isolating biotinylated proteins and another 50 µL for total protein evaluation, the latter of which were combined with 50 µL of 2 x sample buffer (15% glycerol (v/v), 100 mM DTT, 2% SDS (w/v), 0.006% Bromophenol blue (w/v), and 80 mM Tris pH 6.8) and heated to 95^o^C for 5 minutes. The membrane-localized biotinylated protein fractions were isolated from each 100 µL aliquot on columns loaded with 200 µL of 50% slurry of NeurAvidin® Agarose. After a 2 hour incubation at 4^o^C, unbound proteins were eluted by centrifugation and columns washed 3 times with Wash Buffer supplemented with protease inhibitor. Biotinylated proteins were then eluted using 200 µL of SDS-PAGE sample buffer (10% glycerol (v/v), 1% SDS, 50 mM DTT and 62.5 mM Tris pH 6.8) and centrifugation. 15 µL aliquots of each protein sample were separated on two identical 7.5% SDS-PAGE gels; one gel was Coomassie-stained while the other transferred to 0.45 µm PROTRAN® nitrocellulose membrane (Whatman®). After blocking in Tween-20 Tris-buffered saline (TTBS) containing 5% skim milk powder (w/v), the membrane was incubated with α-LCa_v_3 antibody (1:500 in TTBS with 5% milk) overnight a 4^o^C, washed the next day 2 x 15 minutes with TTBS, then incubated with 1:1500 goat α-rabbit HRP (Jackson ImmunoResearch Laboratories, Inc.) at room temperature for 3 hours. Membrane was subsequently washed 3 x 15 min with TTBS, HRP-activated chemiluminescence was detected by exposing the membrane to BioMax Light Film (Kodak), and both the developed film and the Coomassie-stained gel were imaged and densinometric analysis carried out using an AlphaImager HP acquisition system and the AlphaEase® FC software (Alpha Innotec).

**References**

1. Senatore A, Spafford JD (2010) Transient and big are key features of an invertebrate T-type channel (LCav3) from the central nervous system of Lymnaea stagnalis. *J Biol Chem* 285(10):7447-7458.

2. Bimboim HC, Doly J (1979) A rapid alkaline extraction procedure for screening recombinant plasmid DNA. *Nucleic acids research* 7(6):1513-1523.

3. van Moorsel CHM, van Nes WJ, Megens H-J (2000) A quick, simple, and inexpensive DNA extraction protocol for Gastropods. *Malacologia* 42(:203-206.

4. Marois R, Croll RP (1992) Development of serotoninlike immunoreactivity in the embryonic nervous system of the snail Lymnaea stagnalis. *J Comp Neurol* 322(2):255-265.

5. Nagy T, Elekes K (2002) Ultrastructure of neuromuscular contacts in the embryonic pond snail Lymnaea stagnalis L. *Acta Biol Hung.* 53(1-2):125-139.

6. McComb C, Varshney N, Lukowiak K (2005) Juvenile Lymnaea ventilate, learn and remember differently than do adult Lymnaea. *J Exp Biol* 208(Pt 8):1459-1467.

7. Chomczynski P, Sacchi N (1987) Single-step method of RNA isolation by acid guanidinium thiocyanate-phenol-chloroform extraction. *Anal Biochem* 162(1):156-159.

8. Andersen CL, Jensen JL, Orntoft TF (2004) Normalization of real-time quantitative reverse transcription-PCR data: a model-based variance estimation approach to identify genes suited for normalization, applied to bladder and colon cancer data sets. *Cancer Res* 64(15):5245-5250.

9. Pfaffl MW (2001) A new mathematical model for relative quantification in real-time RT-PCR. *Nucleic Acids Res* 29(9):e45.

10. Senatore A, Boone AN, Spafford JD (2011) Optimized transfection strategy for expression and electrophysiological recording of recombinant voltage-gated ion channels in HEK-293T cells. *J Vis Exp* 47(:doi: 10.3791/2314.

11. Chemin J et al. (2002) Specific contribution of human T-type calcium channel isotypes (alpha(1G), alpha(1H) and alpha(1I)) to neuronal excitability. *J Physiol* 540(Pt 1):3-14.

12. Thomas P, Smart TG (2005) HEK293 cell line: a vehicle for the expression of recombinant proteins. *J Pharmacol Toxicol.Methods* 51(3):187-200.

13. Spafford JD, Dunn T, Smit AB, Syed NI, Zamponi GW (2006) In vitro characterization of L-type calcium channels and their contribution to firing behavior in invertebrate respiratory neurons. *J Neurophysiol* 95(1):42-52.
